# Heavy‐Atom Quantum Tunnelling in Spin Crossovers of Nitrenes**

**DOI:** 10.1002/anie.202206314

**Published:** 2022-07-05

**Authors:** Eric R. Heller, Jeremy O. Richardson

**Affiliations:** ^1^ Laboratory of Physical Chemistry ETH Zürich 8093 Zürich Switzerland

**Keywords:** Ab Initio Calculations, Instanton Theory, Nitrenes, Quantum Tunneling, Spin Crossover

## Abstract

We simulate two recent matrix‐isolation experiments at cryogenic temperatures, in which a nitrene undergoes spin crossover from its triplet state to a singlet state via quantum tunnelling. We detail the failure of the commonly applied weak‐coupling method (based on a linear approximation of the potentials) in describing these deep‐tunnelling reactions. The more rigorous approach of semiclassical golden‐rule instanton theory in conjunction with double‐hybrid density‐functional theory and multireference perturbation theory does, however, provide rate constants and kinetic isotope effects in good agreement with experiment. In addition, these calculations locate the optimal tunnelling pathways, which provide a molecular picture of the reaction mechanism. The reactions involve substantial heavy‐atom quantum tunnelling of carbon, nitrogen and oxygen atoms, which unexpectedly even continues to play a role at room temperature.

## Graphical abstract

The spin crossover from triplet to singlet in two nitrene reactions is accompanied by simultaneous heavy‐atom quantum tunnelling. Instanton theory was used to unveil the molecular mechanism of this tunnelling process and, in addition to excellent agreement with experiment, appreciable tunnelling effects were found even at room temperature.

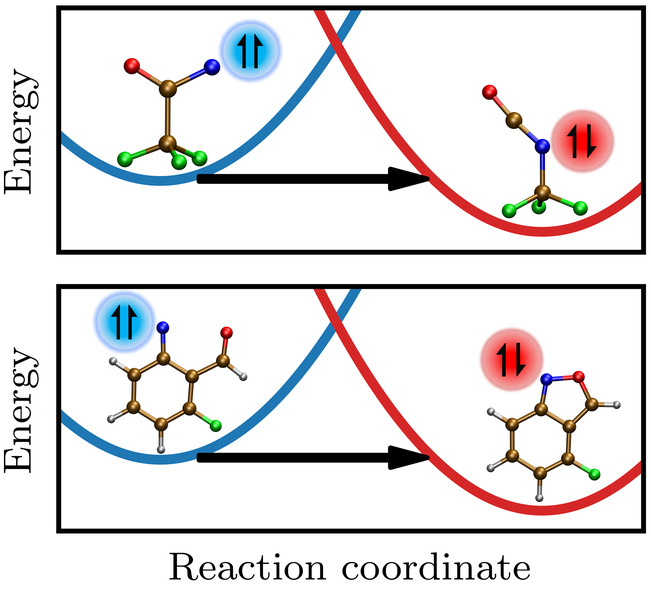

## Introduction

Due to their role as versatile reactive intermediates in several important organic reactions, carbenes and nitrenes are molecules of high interest,^[1]^ not only for synthetic chemistry,[^2^, ^3^, ^4^, ^5^, ^6^, ^7^] but also for coordination chemistry[^8^, ^9^, ^10^, ^11^, ^12^, ^13^] and the functionalization of nanomaterials.[^14^, ^15^, ^16^, ^17^, ^18^] It is thus highly desirable to understand their reactivity in detail. Interestingly, in a number of matrix‐isolation experiments at cryogenic temperatures on carbenes[^19^, ^20^, ^21^, ^22^] and (more recently) for nitrenes,^[23]^ indications of nuclear tunnelling such as temperature‐independent rates and large kinetic isotope effects (KIEs) have been observed. In order to confirm the tunnelling hypothesis and characterize the mechanism, it was crucial that the reactions could be simulated using theoretical approaches.[^24^, ^25^, ^26^, ^27^, ^28^, ^29^, ^30^, ^31^, ^32^]

The feature that makes the reactions cited above amenable to established theoretical methods is that the rate‐determining step takes place adiabatically on a single electronic state, which allows the Born–Oppenheimer approximation to be used. However, due to the two additional non‐bonded electrons on the carbon or nitrogen atom, both carbenes and nitrenes may exist either in their singlet or triplet state, and the spin‐crossover process is nonadiabatic. Three recent studies have presented convincing evidence for nitrene reactions in which the spin crossover is the rate‐determining step. They additionally found that this process is accompanied by tunnelling of hydrogen^[33]^ or heavier atoms.[^34^, ^35^] In order to simulate these nonadiabatic reactions, methods are required which go beyond the Born–Oppenheimer approximation.

Theoretical investigations of spin crossovers commonly start by locating the minimum‐energy crossing point (MECP) of the two spin states. Based on the knowledge of the MECP and the reactant minimum, nonadiabatic transition‐state theory (NA‐TST)[^36^, ^37^, ^38^] can be applied with nuclear tunnelling effects included by the weak‐coupling (WC) approximation.^[39]^ This constitutes the current standard approach for the calculation of spin‐crossover rates.[^33^, ^34^, ^40^, ^41^, ^42^, ^43^, ^44^]

As the name of the WC method suggests, it is based on the assumption of weak spin–orbit coupling between the two states, as for Fermi's golden rule. We will demonstrate that the golden‐rule assumption itself is valid for the nitrene reactions under investigation. However, the WC method additionally relies on a crude linear approximation of the potential‐energy surfaces (PESs) around the MECP. While this approximation would be valid for shallow tunnelling at high temperatures, where the reaction proceeds at an energy that lies only slightly lower than the MECP, its applicability to the description of deep tunnelling at energies close to the reactant zero‐point energy (ZPE) cannot be rigorously justified. In this work, we will show that the WC method fundamentally breaks down and leads to unphysical predictions for the deep tunnelling exhibited by the two nitrene reactions. It is hence evident that new theoretical methods are needed to provide reliable insight into the tunnelling mechanism underlying the experimental results.

For adiabatic reactions, semiclassical instanton theory has become a well‐established method since it finds an excellent balance between a rigorous theoretical foundation and a high computational efficiency.[^31^, ^45^, ^46^, ^47^, ^48^, ^49^, ^50^, ^51^, ^52^, ^53^, ^54^, ^55^, ^56^, ^57^] More recently, a nonadiabatic version of instanton theory has been derived from first principles.[^58^, ^59^] Like the WC method, it is also based on Fermi's golden rule, but importantly treats the full‐dimensional PESs in an ab initio manner. This golden‐rule instanton theory has been shown to be in excellent agreement with exact quantum‐mechanical rates across the whole temperature range[^59^, ^60^, ^61^, ^62^] and can even be extended beyond the golden‐rule limit.^[63]^ Unlike full quantum‐mechanical solutions, which are intractable for all but the simplest systems, instanton theory is computationally amenable to on‐the‐fly simulations of sizeable molecular systems.^[64]^ The central concept of the theory is the optimal tunnelling pathway. Thus, in addition to quantitative predictions of rates and KIEs, it gives detailed mechanistic insight.

The experiments for the two reactions under consideration were carried out at cryogenic temperatures, at which the excited vibrational states are not thermally accessible. Hence, the only mechanism for the reaction to proceed is via nuclear tunnelling out of the vibrational ground state, giving rise to a temperature‐independent plateau of the rate constant in the low‐temperature limit.^[65]^ The convergence of the instanton method becomes slightly more challenging under these conditions, but similar to a tunnelling‐splitting calculation, which is also defined in the low‐temperature limit,[^66^, ^67^, ^68^, ^69^, ^70^, ^71^] the theory is in principle able to capture this important physical case.^[62]^

In this work we will investigate nonadiabatic tunnelling in spin‐crossover processes by considering two specific examples from nitrene chemistry. Although heavy‐atom tunnelling is conventionally thought of as being restricted to cryogenic temperatures, we here unveil the significance of such effects even at room temperature, implying that heavy‐atom tunnelling may surprisingly be relevant under typical reaction conditions of synthetic chemistry.

## Results and Discussion

**Scheme 1:**
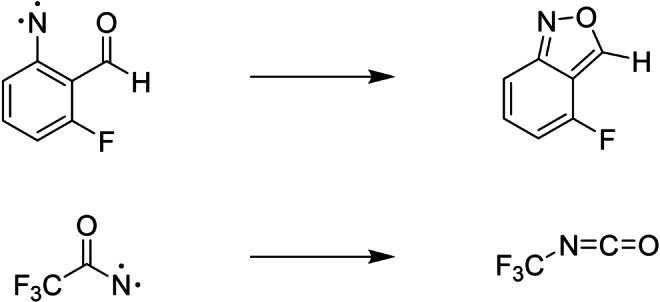
Cyclization (top) and isomerization reaction (bottom).

In this work we simulate the cyclization reaction of a 2‐formylaryl nitrene^[34]^ and the isomerization reaction of trifluoroacetyl nitrene^[35]^ (see Scheme 1). In the experiments, both these nitrenes were prepared in their triplet state and isolated in an inert‐gas matrix at cryogenic temperatures between 2.8 and 23 K. They then underwent spin crossover to the singlet state in addition to the rearrangement of the molecular geometry into the corresponding benzisoxazole and isocyanate. Based on the low‐temperature plateau of the measured rate constants, it was proposed that deep nuclear tunnelling plays a crucial role in these reactions.

In the previous studies[^34^, ^35^] a theoretical analysis of possible reaction pathways was carried out. The results suggested that these reactions belong to the interesting class of processes in which the spin crossover and the nuclear tunnelling occur simultaneously (as opposed to sequentially). In addition, in Ref. [34], rate constants based on the WC approximation were calculated for both reactions. Although at first sight, it appears positive that some of the results are within an order of magnitude of the experimental measurements, the WC method does not capture the low‐temperature plateau observed in experiment. This of course casts doubt on the validity of its results.

The failure of the WC method to capture the physical behaviour of the rate arises from the inherent linear approximation of the potentials around the MECP, which clearly breaks down close to the reactant minimum. The method cannot therefore be expected to give a reasonable description of reactions at very low temperature, where tunnelling takes place dominantly from the vibrational ground state. Moreover, the WC approach is not able to predict possible changes in the reaction mechanism due to multidimensional tunnelling effects such as corner cutting.

In order to gain well‐founded theoretical insight into heavy‐atom tunnelling in the two nitrene reactions, we hence need to go beyond the approximations of previous studies. We pay particular attention to the two main aspects of any practical molecular simulation: i) the accuracy of the electronic structure; ii) the validity of the assumptions underlying the rate theory.

The crucial importance of the electronic structure arises from the sensitive dependence of rate calculations on the quality of the underlying PESs, on which the nuclear dynamics take place. As detailed in the Supporting Information, our investigations of the two systems under consideration showed that an accurate description of dynamic correlation is of particular importance in these reactions, which rules out the validity of the complete active space self‐consistent field (CASSCF) method alone. In this work, we therefore employ state‐of‐the‐art double‐hybrid density‐functional theory (DFT)^[72]^ using the B2‐PLYP functional^[73]^ with dispersion interactions.[^74^, ^75^] Double‐hybrid DFT goes beyond the single‐hybrid DFT used in previous work,[^34^, ^35^] in that not only exact exchange from Hartree–Fock but also correlation from second‐order perturbation theory is included. This method is expected to give an accurate description of the two nitrene reactions, since it has been shown to give excellent results for a host of electronically very similar carbenes.^[76]^ These calculations are supplemented by further computations with multireference Møller–Plesset perturbation theory to second order (MRMP2).^[77]^ MRMP2 is very similar to the better known CASPT2 method, but constructs the first‐order wavefunction in a slightly different way.^[78]^ In the case of the isomerization reaction, it was possible to optimize the reactant minimum and MECP with single‐state MRMP2(10,9), while the significantly larger molecular system in the cyclization reaction only allowed single‐point MRMP2(10,10) calculations at the DFT‐optimized configurations. For both reactions, we found that the multiconfigurational character of the electronic wavefunction at the minimum and MECP is relatively small, which is confirmed by an increase of the MRMP2 barrier heights compared to the DFT results by only 12 % and 11 %. This reasonable agreement between the two methods confirms the validity of double‐hybrid DFT for these reactions, at least for locating the tunnelling pathways. For improved quantitative accuracy, a small correction can be applied based on the difference with the MRMP2 result, as explained below.

We can go beyond the WC approximation of the rate using semiclassical golden‐rule instanton theory, which provides an accurate description of nuclear quantum effects such as ZPE and multidimensional tunnelling.[^58^, ^59^, ^62^] In contrast to Fermi's golden rule, which formally requires the computation of wavefunction overlaps between vibronic states,^[79]^ instanton theory is rooted in the path‐integral formulation of quantum mechanics. Here, the path integral is dominated by two classical trajectories travelling on the triplet and singlet surfaces, which join smoothly together at the so‐called “hopping point”. These trajectories obey Newton's equations of motion in imaginary time,^[80]^ which enables them to travel below the barrier (in the classically forbidden region) and therefore capture nuclear tunnelling in an intuitive manner.[^58^, ^59^] The amount of imaginary time spent on the singlet state is τ and on the triplet state is βℏ-τ , such that the overall time is related to the inverse temperature, $\beta =1/k_{B}T.$ Together, these trajectories define the optimal tunnelling pathway, known as the “instanton”.

The key computational step is the optimization of this instanton pathway (including finding the optimal τ ), which is facilitated by discretizing the trajectories in the form of a ring polymer.[^62^, ^81^] In this way, the problem can be turned into a standard saddle‐point search of the ring polymer, based on potentials, gradients and Hessians computed on‐the‐fly with double‐hybrid DFT.

In the golden‐rule limit, the semiclassical instanton (SCI) expression for the rate constant is then given by Equation 1,^[62]^

(1) $$k_{SCI}\left(T\right)=\sqrt{2\pi }\;\frac{\beta \Delta^{2}}{\hbar }\;\frac{Z^{\neq }}{Z_{T}}\;e^{-S/\hbar }\;$$

where Δ is the spin–orbit coupling measured at the hopping point, S is the instanton action, given as the sum of the classical actions of the individual states, and $Z^{\neq }$ is the instanton partition function, which like the reactant (triplet) partition function $Z_{T}$ contains translational, rotational and vibrational contributions for each degree of freedom. Note that the vibrational component of $Z^{\neq }$ is computed from the second derivatives of the action with respect to the ring‐polymer beads and imaginary time, τ .^[62]^ The expression in Equation (1) constitutes a generalization of transition‐state theory. The key difference is that the exponential dependence on the classical activation energy is replaced by S/ℏ , which typically increases the rate by accounting for tunnelling effects.

**Figure 1:**
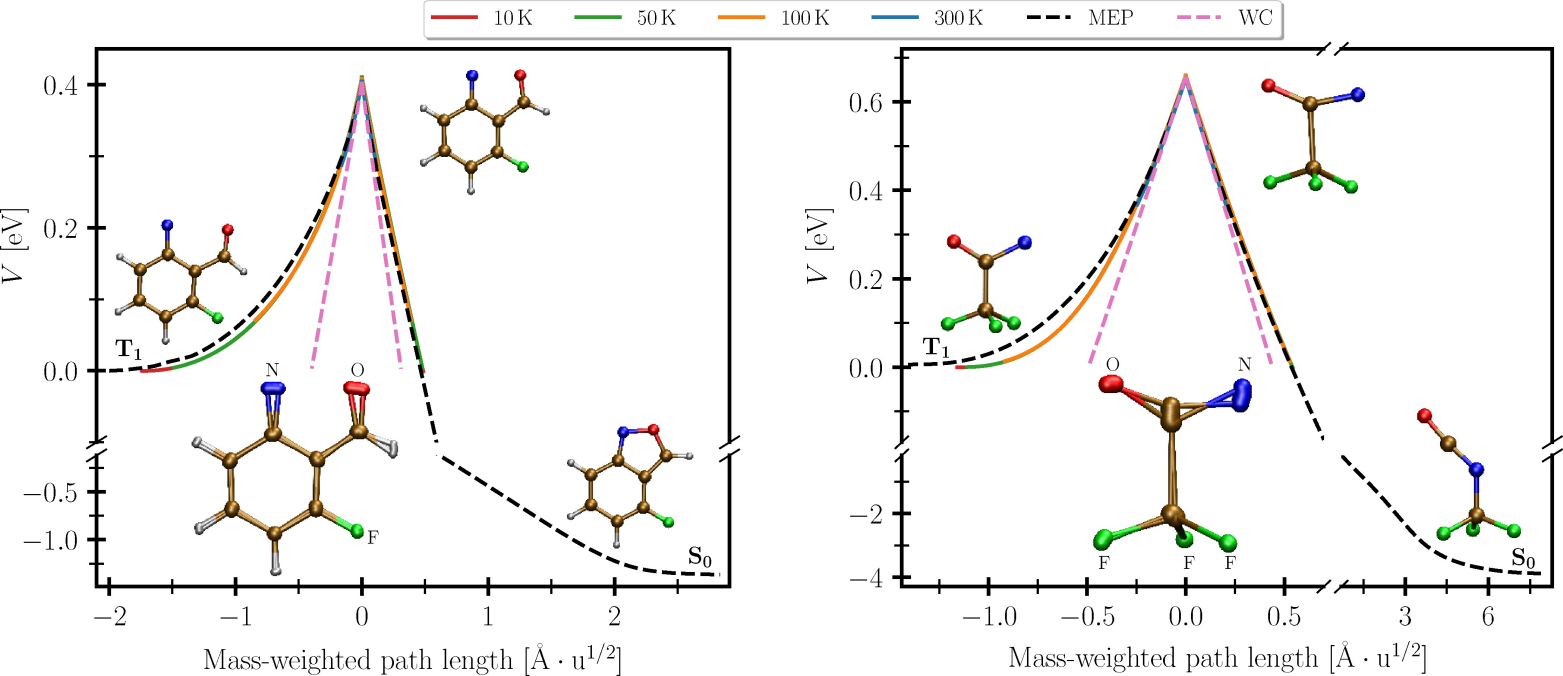
Potential energy computed with double‐hybrid DFT along the MEPs and instanton pathways at various temperatures as well as the linear WC approximation of the PESs around the MECP for the cyclization (left) and isomerization reaction (right). The insets below the barriers depict the change in the molecular structure along the instantons in the low‐temperature limit. All atoms except for the carbons and hydrogens are labelled. Bonds are drawn for the structures at the turning points of the path. The structures of the triplet and singlet minima are shown in the respective wells and the MECP structure is illustrated near the barrier top.

In Figure 1, we depict the potential‐energy profile along the instanton pathways of both the cyclization and isomerization reaction. The instanton is based on a least‐action principle and thus rigorously defines an optimal tunnelling pathway which constitutes the key step of the reaction mechanism. For comparison, the mass‐weighted minimum‐energy pathways (MEPs) and the linear approximation to the potentials as employed by the WC method are also shown. Notice the striking misrepresentation of the PESs inherent in the WC approximation at low energies. We will later explore the effects of this on the predicted rates.

From Figure 1 it can be seen that in both cases the reactant and product minima lie on opposite sides of the crossing seam and are separated by a cusped barrier. This is analogous to the normal regime of Marcus theory. The insets below the barrier in Figure 1 illustrate the instanton pathways at 10 K. For both the cyclization and isomerization reaction the instantons exhibit considerable delocalization indicating substantial heavy‐atom tunnelling of nitrogen, oxygen and carbon atoms.

It can be seen in the cyclization reaction that the oxygen and nitrogen atoms tunnel toward one another in order to complete the isoxazole five‐membered ring. The oxygen atom contributes 48 % of the squared mass‐weighted tunnelling path length (SMWTPL), closely followed by nitrogen with 35 %.

The instanton pathway for the isomerization reaction reveals that the bottleneck is the cleavage of the C−C bond, which is overcome by means of heavy‐atom tunnelling. After emerging on the product side of the barrier, the NCO group shifts over to form the C−N bond of the isocyanate product. In this reaction the dominant contribution to the SMWTPL comes from the carbon and nitrogen atoms in the NCO group with 43 % and 34 %. The second carbon and the oxygen atom contribute 7 % and 9 % to the SMWTPL, while the fluorine atoms account for only 7 % in total. It had previously been proposed that the CF_3_ group was responsible for the tunnelling.^[35]^ Our simulations show that in fact it is the NCO group that tunnels, whereas the shift of the CF_3_ group from the carbon to the nitrogen atom takes place after the barrier crossing.

From the knowledge of the instanton pathways, the rate constants can now be computed using Equation (1). However, in order to effectively account for missing multiconfigurational effects not captured by DFT, we first scaled the potential energies (relative to the reactant minimum) of the MECP and along the MEPs and instanton pathways by the ratio of the MRMP2 and DFT barrier height. This is a common trick to improve the energetics, when higher‐level methods are too computationally expensive for structure optimizations.[^34^, ^69^, ^82^, ^83^, ^84^, ^85^, ^86^] Note that this procedure is only valid if the correction constitutes a small change to a PES that is already described reasonably well. The correction of the instanton results based on the potential energy at the MECP is justified here because the instanton pathways remain close to the MEPs, as can be seen from Figure 1, and the difference between the DFT and MRMP2 barrier heights is not dramatic.

**Figure 2:**
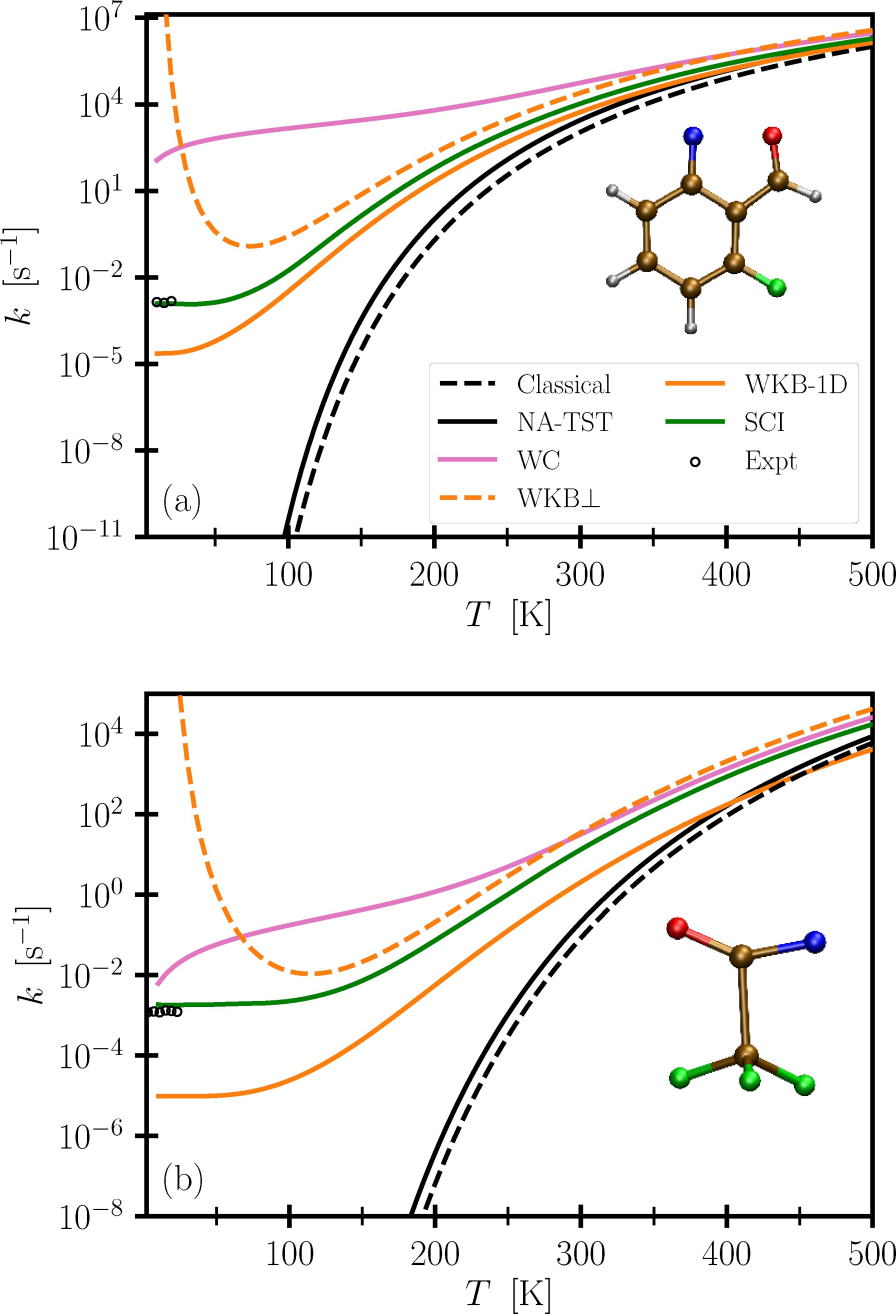
Reaction rate constants for the cyclization (top) and isomerization reaction (bottom) computed with various methods, including semiclassical golden‐rule instanton theory (SCI), and compared to experiment.[^34^, ^35^] All calculations have been corrected with the MRMP2 barrier height as described in the text; the uncorrected results can be found in the Supporting Information. The insets show the molecular structure at the MECP of the respective reaction.

In Figure 2, we present the instanton results as well as those from various other methods. From the Figure it becomes evident that instanton theory is in excellent agreement with experiment for both reactions. Together with the analysis of the pathways above, this provides further confirmation that it is indeed heavy‐atom tunnelling that is observed in experiment.

At high temperatures the WC rate constants are in good agreement with the instanton results. In fact, formally both WC and instanton theory have the same correct classical limit equal to NA‐TST. However, at temperatures below 300 K, the WC method overestimates the rate by orders of magnitude compared to experiment and instanton theory. Note that our WC results are different from the ones reported in Ref. [34], which used a lower level of DFT leading to a barrier (including ZPE) for the cyclization reaction which is 46 % higher than ours. The error compensation from a barrier that is too high, due to the approximate electronic structure, and too narrow, due to the linear approximation in WC, explains why their WC results are fortuitously closer to the experimental rate constants than the WC calculations reported here. More importantly though, Figure 2 shows that the WC approximation leads to qualitatively wrong results. It is well known that due to nuclear tunnelling the rate constant of unimolecular reactions should reach a plateau at low temperatures, as is correctly described by instanton theory.[^62^, ^65^] The WC method cannot, however, capture this plateau and is thus inappropriate for the description of cryogenic matrix‐isolation experiments.

The one‐dimensional Wentzel–Kramers–Brillouin (WKB) approximation, which accounts for tunnelling along the MEPs,[^87^, ^88^] can also be used to approximate Fermi's golden rule and does predict a low‐temperature plateau. One might expect a good agreement with the instanton results due to the similarity between the instanton pathways and the MEPs depicted in Figure 1. The 1D WKB results deviate, however, from both instanton theory and experiment by orders of magnitude. This is because they neglect the ZPE effects from the modes orthogonal to the MEPs. It is not trivial to include these effects, as can be seen from the WKB⊥ curves, where, in analogy to Eyring transition‐state theory, the orthogonal modes have been included as quantum harmonic oscillators with frequencies corresponding to the MECP and reactant minimum (as in NA‐TST) leading to an unphysical divergence of the rate at low temperatures. Instanton theory does not assume separability between the reaction coordinate and the orthogonal degrees of freedom and thus captures the dependence of the ZPE along the multidimensional tunnelling path.

We can leverage the accuracy of instanton theory to study the reactions at temperatures where the nitrenes react too quickly to isolate them and measure a rate. While it is expected that tunnelling is the key mechanism for a reaction to proceed at low temperatures, we surprisingly find that even at 300 K nuclear quantum effects continue to speed up the rate of the cyclization and isomerization by factors of 10 and 160 compared to the (fully) classical case. Further comparison to the NA‐TST rate (which includes ZPE but not tunnelling effects) reveals that heavy‐atom tunnelling alone accounts for speed‐ups of 4 and 60.

Although hydrogen‐atom tunnelling is not particularly unusual, and a number of examples of heavy‐atom tunnelling have been reported at cryogenic temperatures,[^28^, ^29^, ^89^, ^90^] such strong effects from heavy‐atom tunnelling are thought to be very rare at room temperature.^[91]^ For adiabatic reactions, a simple rule of thumb states that significant tunnelling only occurs below a *crossover* temperature $T_{c}={\hbar \omega }_{b}/2\pi k_{B}$ which depends on the curvature $\omega_{b}$ of the barrier top.^[65]^ The behaviour of reactions in the nonadiabatic limit is, however, fundamentally different because the barrier top is cusped, as can be seen in Figure 1. In the high‐temperature limit, the instanton is found close to the barrier top. In this case, a simple tunnelling factor of the form ${\exp\left(T_{o}/T\right)}^{3}$ can be derived,^[58]^ where $T_{o}^{3}=\frac{\hbar^{2}}{24mk_{B}^{3}}{\left(\frac{\kappa_{T}\kappa_{S}}{\kappa_{T}-\kappa_{S}}\right)}^{2}$ is defined in terms of the slopes $\kappa_{T}$ and $\kappa_{S}$ of the electronic states at the crossing point.^[92]^ In the nonadiabatic case, there is no formal crossover from shallow to deep tunnelling, but rather a smooth transition. We thus suggest the interpretation of $T_{o}$ as an *onset* temperature below which tunnelling starts to become important. Due to the cubic dependence on $T_{o}/T$ inside the exponential of the tunnelling factor, the significance of nuclear tunnelling will rapidly increase below this temperature.

For the cyclization and isomerization reactions, we obtain onset temperatures of 434 K and 514 K, implying that carbon, nitrogen and oxygen atoms can tunnel even above room temperature. This is in stark contrast to adiabatic reactions, for which the crossover temperature is rarely much higher than 300 K for hydrogen‐tunnelling reactions and typically much lower for heavy‐atom rearrangements, implying that heavy‐atom tunnelling is not significant at room temperature. Note however that the simple tunnelling factor stated above can only be used for a rough assessment about whether tunnelling plays a role in a given reaction. This is due to the linear approximation also inherent in the WC method, which as shown in Figure 2 already starts to break down at 300 K. Quantitative predictions below the onset temperature require using instanton theory.

Although this analysis makes it clear that tunnelling is more likely to be important for nonadiabatic reactions than for adiabatic reactions, it is still a rather surprising finding that there is significant tunnelling of heavy atoms at typical laboratory conditions, especially as these reactions are in the Marcus normal regime. While tunnelling is known to be common in the inverted regime from the related field of electron transfer,[^79^, ^93^, ^94^] reactions in the normal regime are typically expected to exhibit smaller tunnelling effects (unless they involve H‐atom transfers).^[95]^ This can be understood from the definition of the onset temperature above. In the normal regime (which has a cusped intersection), the two gradients are antiparallel at the crossing point and thus $\kappa_{T}$ and $\kappa_{S}$ have opposite signs, whereas in the inverted regime (which has a sloped intersection), the gradients are parallel and hence have the same sign. This implies that the onset temperature, which depends inversely on $\left|\kappa_{T}-\kappa_{S}\right|$ , will typically be higher in the inverted regime and therefore that it is more likely to find heavy‐atom tunnelling at room temperature. Additionally, the tunnelling effects in the inverted regime tend to be larger because the instanton action associated with the propagation on the product state contributes with a negative sign leading to a reduced value of S , while in the normal regime both actions are positive leading to a slower rate.^[59]^ In previous work, we argued that this was the cause of the large heavy‐atom tunnelling factors of the spin crossover in thiophosgene, which occurs in the inverted regime.^[64]^ In this work, we demonstrate that reactions in the normal regime can also have substantial heavy‐atom tunnelling, as long as the magnitudes of the slopes $\left|\kappa_{T/S}\right|$ are large enough.

It is common to measure KIEs as a powerful experimental approach to obtain insight into tunnelling reactions. Therefore, we also computed the ^14^N/^15^N KIE for both reactions using two independent instanton calculations. Here we discuss only the low‐temperature limit; predictions at higher temperatures can be found in the Supporting Information. Our result of 1.35 for the isomerization reaction is in excellent agreement with the range of experimental values 1.18–1.44^[35]^ and thus confirms the exceptionally strong nitrogen tunnelling effects evident from the instanton trajectory depicted in the inset of Figure 1.^[96]^ We furthermore predict a ^14^N/^15^N KIE of 1.4 for the cyclization reaction, which could be verified by future experiments.

Our analysis of the instantons above revealed that the NCO‐carbon in the isomerization and the oxygen atom in the cyclization contribute even more to the tunnelling pathway than the nitrogens. We hence computed the ^12^C/^13^C and ^16^O/^18^O KIEs in the low‐temperature limit for the respective reactions and predict even larger values of 1.8 and 2.4. In this case we used a simple approximate scheme by assuming that the instanton pathway would not change significantly upon isotopic substitution.^[97]^ In the Supporting Information, we show that the dominant effect on the rate is due to the change in the value of the instanton action and not the path. This not only validates the assumption but also indicates that the KIE is dominated by tunnelling effects and not a change in ZPE.

## Conclusion

We have studied the effects of heavy‐atom tunnelling on low‐temperature spin‐crossover reactions of two nitrenes and obtained quantitative agreement with experimental rate constants. To achieve this level of accuracy, it was necessary to employ MRMP2 calculations on top of double‐hybrid DFT in order to obtain an adequate description of the PESs. However, even with an accurate description of the electronic structure, meaningful results can only be attained with a state‐of‐the‐art rate theory such as the golden‐rule instanton formalism.

Our results highlight the shortcomings of the commonly used[^33^, ^34^, ^38^, ^40^, ^41^, ^42^, ^43^, ^44^] WC approximation to describe quantum tunnelling at low temperatures. Although the WC method can indeed give an indication of when tunnelling becomes important, it is not at all reliable for quantitative predictions away from the classical limit.

In contrast, the high accuracy of golden‐rule instanton theory demonstrated in this work and in several other studies[^60^, ^61^, ^62^, ^64^] endows the method with high predictive power. It resolves the fundamental problems of WC, and we thus recommend it becomes the new standard method for simulations of spin‐crossover rates in which tunnelling plays a role (i.e. whenever $T<T_{o}$ ).

Based on the instanton pathways, we can obtain detailed insight into the rearrangement mechanism. We unveiled substantial amounts of heavy‐atom tunnelling in the reactions of both nitrenes. In particular, we demonstrated the origin of the unprecedentedly large ^14^N/^15^N KIE measured in the isomerization reaction, and in addition, predicted other large nitrogen, carbon and oxygen KIEs, which we hope to be confirmed by future experiments. In future studies, the predictive power of instanton theory could be used to investigate whether these subtle effects can provide tunnelling control of chemical reactions by means of isotopic substitution.[^24^, ^25^, ^26^]

While in the inverted regime, tunnelling drastically changes the reaction mechanism due to strong corner cutting,^[64]^ it was not obvious that such behaviour would carry over into the normal regime (as in the two reactions studied in this work). Although there was very little corner cutting, we nonetheless found significant speed‐ups of the two nitrene reactions even at room temperature. The reason for such unexpectedly substantial heavy‐atom tunnelling at room temperature is the narrow, cusped barrier between the diabatic states, which is in stark contrast to the rounded barrier tops encountered in typical adiabatic reactions. We thus expect tunnelling to be generally more important in nonadiabatic reactions, which commonly have narrow barriers, than for adiabatic reactions. Our findings therefore hint at the possibility that nuclear quantum effects might be more prevalent in the reactivity of nitrenes (and possibly other organic compounds) than typically presumed.

While the reactions considered in this work take place in inert‐gas matrices, the description of synthetic chemistry would have to include solvent effects. Although instanton theory cannot simulate liquids explicitly, solvent effects can be approximately accounted for by means of polarizable continuum models, methods based on the spectral density of the bath^[61]^ or by building small clusters that represent the first solvent shells. In this way, instanton theory could be used to search for tunnelling effects in chemical reactions under standard wet‐lab conditions.

## Conflict of interest

The authors declare no conflict of interest.

1

## Supporting information

As a service to our authors and readers, this journal provides supporting information supplied by the authors. Such materials are peer reviewed and may be re‐organized for online delivery, but are not copy‐edited or typeset. Technical support issues arising from supporting information (other than missing files) should be addressed to the authors.

Supporting Information

Supporting Information

Supporting Information

Supporting Information

Supporting Information

Supporting Information

Supporting Information

Supporting Information

Supporting Information

Supporting Information

Supporting Information

## Data Availability

The data that support the findings of this study are available in the Supporting Information of this article.
