# Supplementary material for: Heavy‐Atom Quantum Tunnelling in Spin Crossovers of Nitrenes
Source: Angew Chem Int Ed Engl. 2022 Jul 5;61(33):e202206314. doi: 10.1002/anie.202206314 (PMC9540336; doi:10.1002/anie.202206314)
Supplement: Supplementary file 7 — Supporting Information [file ANIE-61-0-s007.pdf]

Angewandte Chemie
Eine Zeitschrift der Gesellschaft Deutscher Chemiker

Supporting Information

Heavy-Atom Quantum Tunnelling in Spin Crossovers of Nitrenes

*E. R. Heller, J. O. Richardson**

Supporting Information

Contents

1 Computational Methods 1

2 Rate calculations 2

3 Ring-polymer instanton results 6

4 Collected results 11

References 13

1 Computational Methods

Unless otherwise stated, the optimizations and rate calculations in this work are based on potential-energy surfaces computed with double-hybrid density-functional theory (DFT). We employ the B2-PLYP functional[1] using the resolution-of-the-identity (RI) approximation for the perturbation-theory calculation[2] with corresponding auxiliary basis set[3] as implemented in the ORCA 4 program suite.[4,5] In the self-consistent field cycle, the RI-J approximation is used for the Coulomb integrals while the Hartree–Fock (HF) exchange is evaluated with COSX numerical integration. Since double-hybrid functionals can be rather sensitive to the choice of integration grid, we select the finest grids available in ORCA 4.0 for both the DFT and HF-exchange calculation.

Dispersion interactions are accounted for with Grimme's DFT-D3 correction[6] with Becke–Johnson damping.[7] In order to accurately describe the nitrene's non-bonded electrons, we choose the Karlsruhe triple-ζ valence basis set with polarization and additional diffuse functions (def2-TZVPD)[8] in conjunction with the corresponding auxiliary basis sets for the calculations based on the RI approximation.[9,10]

While analytic gradients are available for double-hybrid functionals, the Hessians required for the rate calculations are computed numerically by central finite differences.

We treat the molecules in vacuum under the assumption that the interaction with the Ar, Ne or N_2 gas matrices used in experiment is weak. This seems reasonable because the experimentally measured rate constants do not differ by more than a factor of 2 between the different gases.

The MECPs were obtained by a constrained minimization on the crossing seam using a Lagrange multiplier.[11] For the ring-polymer instanton optimization[12] we employed an eigenvector-following algorithm.[13–15] In order to avoid the recomputation of the Hessian at each step, we employed a Powell update algorithm.[16]

All structure optimizations of minima and MECPs were converged to a maximum total force of $3 \times 10^{-4}\, E_\mathrm{h}\,\mathrm{\AA}^{-1}$. For the instantons the forces were computed as the negative combined gradient with respect to bead positions and imaginary time τ of the N-bead ring-polymer potential, $U_N/N = S/\beta\hbar$ and converged to a maximum total force of $3 \times 10^{-5}\, E_\mathrm{h}\,\mathrm{\AA}^{-1}$. The τ-coordinate was scaled by a factor $\xi/\beta\hbar$ with ξ in the range of $1 - 5\,\mathrm{\AA}$.

In order to test for the effect of static cor-

relation in the isomerization reaction, we carried out calculations based on multireference Møller–Plesset perturbation theory to second order (MRMP2),[17,18] as implemented in the GAMESS (US) program suite.[19–21] The active space consists of 10 electrons in 9 orbitals. The latter were chosen as the natural orbitals with occupation numbers between 1.98 and 0.02 at the MECP. For these calculations the Karlsruhe triple-ζ valence basis set with polarization (TZVP) was employed. Gradients were computed numerically by central finite differences.

Since optimizations on the MRMP2 level were computationally unfeasible for the larger molecular system of the cyclization reaction, we simply carried out single-point MRMP2 calculations with an active space of 10 electrons in 10 orbitals at the optimized DFT geometries for the reactant and MECP.

The barrier height, $V^{\ddagger}$, is defined as the potential-energy difference between the MECP and the reactant minimum. Results for barrier heights calculated with DFT and CASSCF are presented in Tables SI.1 and SI.2, together with the barrier heights obtained from MRMP2 and CASSCF calculations at the DFT geometries.

For the isomerization we additionally give the barrier heights resulting from the MRMP2 optimization. For both reactions CASSCF overestimates the MRMP2 barrier height by a substantial amount, implying that an accurate description of dynamic correlation is paramount in these reactions. In contrast, DFT (which includes dynamic but not static correlation) underestimates the MRMP2 barrier by no more than 15%, which indicates that the PESs are reasonably well-described by double-hybrid DFT. In order to account for the missing multiconfigurational effects (at least approximately) in our rate calculations, we scale the potential energies by this ratio. Within the classical, NA-TST and WC methods, this means that we simply use the MRMP2 barrier height. For the WKB method, all potentials along the MEPs (obtained with DFT) are scaled by the ratio of the MRMP2 and DFT barrier, while for instanton theory, the same is done for the potentials along the instanton pathways, which then enter into the calculation of the action, S.[12]

Table SI.1: Data at the MECP and reactant minimum of the cyclization reaction relevant for the rate calculations. The rotational constants are given as their geometric mean $(ABC)^{1/3}$. In addition to the DFT and CASSCF(10,10) barriers, we also give the barrier heights computed with MRMP2(10,10) and CASSCF(10,10) from the difference in triplet energies at the DFT geometries (MRMP2//DFT and CASSCF//DFT).

$V^{\ddagger}_{\mathrm{DFT}}$	$0.4034\,\mathrm{eV} = 3254\,\mathrm{cm}^{-1}$
$V^{\ddagger}_{\mathrm{MRMP2//DFT}}$	$0.4575\,\mathrm{eV} = 3690\,\mathrm{cm}^{-1}$
$V^{\ddagger}_{\mathrm{CASSCF//DFT}}$	$0.7338\,\mathrm{eV} = 5918\,\mathrm{cm}^{-1}$
$V^{\ddagger}_{\mathrm{CASSCF}}$	$0.9962\,\mathrm{eV} = 8035\,\mathrm{cm}^{-1}$
Δ	$33.6\,\mathrm{cm}^{-1}$
$\kappa_{\mathrm{T}}/\sqrt{m}$	$0.9766\,\mathrm{eV}/\mathrm{\AA u}^{1/2}$
$\kappa_{\mathrm{S}}/\sqrt{m}$	$-1.2466\,\mathrm{eV}/\mathrm{\AA u}^{1/2}$
λ	0.4391
$(ABC)^{1/3}_{\mathrm{MECP}}$	$4.572 \cdot 10^{-2}\,\mathrm{cm}^{-1}$
$(ABC)^{1/3}_{\mathrm{T}}$	$4.427 \cdot 10^{-2}\,\mathrm{cm}^{-1}$

Since spin–orbit coupling calculations with double-hybrid functionals are currently not implemented in ORCA, we employed the TPSSh[22] functional for its calculation with otherwise identical parameters.[23] We obtained values of $33.6\,\mathrm{cm}^{-1}$ and $23.2\,\mathrm{cm}^{-1}$ at the MECPs for the cyclization and isomerization reaction. The former is in good agreement with previous work.[24] Since there is little corner cutting taking place, the instanton hopping point is practically identical to the MECP for all temperatures, and thus the spin–orbit coupling, Δ, at the MECP was used for the calculation of all instanton rates.

2 Rate calculations

In addition to the rate constants computed with golden-rule instanton theory, the full details of which can be found in Ref. 12, we also present results calculated with classical rate theory, NA-TST, and the WC and WKB approximations. All of these methods require the location of the reactant minimum and MECP of the reactions. We therefore collect the relevant

Table SI.2: Data at the MECP and reactant minimum of the isomerization reaction relevant for the rate calculations. The rotational constants are given as their geometric mean $(ABC)^{1/3}$. In addition to the DFT, MRMP2(10,9) and CASSCF(10,9) barriers, we also give the barrier heights computed with MRMP2(10,9) and CASSCF(10,9) from the difference in triplet energies at the DFT geometries (MRMP2//DFT and CASSCF//DFT).

$V^{\ddagger}_{\mathrm{DFT}}$	$0.6586\,\mathrm{eV}$	=	$5312\,\mathrm{cm}^{-1}$
$V^{\ddagger}_{\mathrm{MRMP2//DFT}}$	$0.7585\,\mathrm{eV}$	=	$6118\,\mathrm{cm}^{-1}$
$V^{\ddagger}_{\mathrm{MRMP2}}$	$0.7383\,\mathrm{eV}$	=	$5955\,\mathrm{cm}^{-1}$
$V^{\ddagger}_{\mathrm{CASSCF//DFT}}$	$0.9930\,\mathrm{eV}$	=	$8009\,\mathrm{cm}^{-1}$
$V^{\ddagger}_{\mathrm{CASSCF}}$	$1.2601\,\mathrm{eV}$	=	$10\,164\,\mathrm{cm}^{-1}$
Δ	$23.2\,\mathrm{cm}^{-1}$		
$\kappa_{\mathrm{T}}/\sqrt{m}$	$1.3403\,\mathrm{eV}/\mathrm{\AA u}^{1/2}$		
$\kappa_{\mathrm{S}}/\sqrt{m}$	$-1.4921\,\mathrm{eV}/\mathrm{\AA u}^{1/2}$		
λ	0.4732		
$(ABC)^{1/3}_{\mathrm{MECP}}$	$9.414 \cdot 10^{-2}\,\mathrm{cm}^{-1}$		
$(ABC)^{1/3}_{\mathrm{T}}$	$9.294 \cdot 10^{-2}\,\mathrm{cm}^{-1}$		

data computed at these points for the cyclization and isomerization reactions in Tables SI.1 and SI.2 and depict the corresponding molecular structures in Fig. SI.1. From these structures it is evident that the planar symmetry is retained throughout the cyclization reaction. In the isomerization reaction both the triplet minimum and the MECP have point group C_1. Thus, there are no symmetry-equivalent reaction pathways in either case.

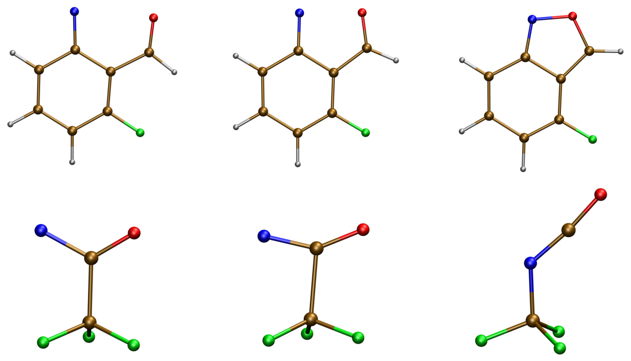

Figure SI.1: From left to right, we depict the T_1 minimum, the MECP and the S_0 minimum of the cyclization (top) and isomerization reaction (bottom). The corresponding xyz-files are included in the supplementary material.

The Lagrange parameter λ stems from the constrained optimization of the MECP and is defined as $\lambda = \kappa_{\mathrm{T}}/(\kappa_{\mathrm{T}} - \kappa_{\mathrm{S}})$ in terms of the signed norms of the mass-weighted gradients on the triplet and singlet states, $\kappa_{\mathrm{T/S}} = \pm||\nabla V_{\mathrm{T/S}}||$. The sign of these norms is chosen such that their product $\kappa_{\mathrm{T}}\kappa_{\mathrm{S}}$ is positive if the gradients are parallel and negative if they are antiparallel. Thus, one can equivalently write $\lambda = \pm|\kappa_{\mathrm{T}}|/(|\kappa_{\mathrm{S}}| \pm |\kappa_{\mathrm{T}}|)$, where positive signs are used in the normal regime, where the two gradients at the MECP are antiparallel, and negative signs in the inverted regime, where the gradients at the MECP are parallel.[12] Note that we mass-weight each atomic coordinate vector x_a by the corresponding atomic mass m_a in the form $\sqrt{\frac{m_a}{m}}x_a$, such that each nuclear degree of freedom has the same reference mass m, which can be chosen arbitrarily. The gradient operator, ∇, is defined by the derivatives with respect to these mass-weighted coordinates.

The Lagrange parameter can then be used to define the effective mass-weighted Hessian $\tilde{\mathsf{H}} = (1-\lambda)\mathsf{H}_{\mathrm{T}} + \lambda\mathsf{H}_{\mathrm{S}}$ in terms of the mass-weighted Hessian matrices of the triplet and singlet states, $\mathsf{H}_{\mathrm{T/S}} = \nabla^2 V_{\mathrm{T/S}}$, at the MECP. After projecting out rotations, translations and the reaction coordinate, one thus obtains the effective frequencies at the MECP, $\tilde{\omega}_k$, as the square root of the eigenvalues of $\tilde{\mathsf{H}}/m$.[25] There are $N_{\mathrm{vib}} = 3N_{\mathrm{atom}} - 6$ vibrational modes with corresponding frequencies ω_k of the reactant, where N_{atom} is the number of atoms in the molecule. Compared to the reactant minimum, the number of vibrations at the MECP is reduced by one ($3N_{\mathrm{atom}} - 7$) because the reaction coordinate has been projected out. The vibrational frequencies at the minima and MECPs for the two reactions with ^{14}N as well as their isotopically substituted analogues with ^{15}N are collected in Tables SI.3 and SI.4.

The lowest frequency occurring at the minimum and MECP geometry of the isomerization reaction roughly corresponds to the rotation of the CF_3 group. In order to check whether this low-frequency is adequately approximated by a

Table SI.3: Vibrational frequencies in cm^{-1} for the reactant minimum and MECP (computed from the eigenvalues of $\tilde{\mathsf{H}}$) of the cyclization reaction with ^{14}N and ^{15}N.

	Triplet minimum						MECP					
^{14}N	88	160	199	224	284	321	–	139	203	227	268	322
	408	492	497	520	609	612	464	488	498	537	608	618
	708	725	812	835	914	1018	702	742	792	808	872	882
	1024	1055	1091	1152	1183	1249	982	1003	1069	1086	1200	1266
	1287	1323	1433	1435	1477	1588	1279	1341	1350	1400	1463	1540
	1619	1772	2990	3208	3225	3232	1574	1633	3048	3200	3233	3236
^{15}N	87	159	198	223	283	318	–	138	202	227	267	321
	402	488	497	519	609	611	462	484	497	535	608	617
	708	722	812	835	914	1018	702	737	792	799	870	882
	1024	1051	1091	1149	1183	1248	982	1003	1066	1085	1200	1266
	1281	1323	1433	1435	1477	1588	1278	1340	1349	1391	1463	1539
	1618	1772	2990	3208	3225	3232	1574	1633	3048	3200	3233	3236

Table SI.4: Vibrational frequencies in cm^{-1} for the reactant minimum and MECP (computed from the eigenvalues of $\tilde{\mathsf{H}}$) of the isomerization reaction with ^{14}N and ^{15}N.

	Triplet minimum					MECP				
^{14}N	36	222	231	359	423	–	17	256	260	327
	509	570	608	713	780	417	526	578	659	734
	1082	1175	1220	1301	1692	882	1135	1227	1248	1642
^{15}N	36	220	230	355	421	–	17	255	259	325
	508	569	600	712	778	413	526	577	658	733
	1074	1175	1220	1300	1692	872	1130	1227	1247	1642

harmonic oscillator as opposed to a hindered rotor, we scanned the potential-energy profile along the rotation around the C–C bond in the reactant minimum (Fig. SI.2). Note that the profile does not exhibit the three-fold symmetry one might expect since the three C–F bonds do not have exactly the same length as we perform this rigid rotation. In principle, one could instead perform a relaxed scan, but this is not expected to dramatically affect the barrier height. Considering that the harmonic frequency of this mode is only $36\,\mathrm{cm}^{-1}$ compared to the barrier height of $258\,\mathrm{cm}^{-1}$, this confirms the validity of the harmonic-oscillator approximation of the mode, at least at the cryogenic temperatures at which the experiments are conducted.

The effect of the molecular rotations is included within the rigid-rotor approximation, where the rotational constants are given by A, B and C.

The NA-TST rate constant is given by[25–28]

$$k_{\text{NA-TST}}(\beta) = \sqrt{\frac{2\pi m}{\beta\hbar^2}}\,\frac{\Delta^2}{\hbar|\kappa_\mathrm{T}-\kappa_\mathrm{S}|}\,\frac{Z^\ddagger}{Z_\mathrm{T}}\,\mathrm{e}^{-\beta V^\ddagger}, \tag{SI.1}$$

where Z_T and $Z^\ddagger$ are the quantum-mechanical partition functions of the reactant and MECP in the usual rigid-rotor, harmonic-oscillator approximation, i.e. using

$$Z_{\mathrm{T,vib}} = \prod_{k=1}^{N_\mathrm{vib}} \frac{1}{2\sinh\frac{1}{2}\beta\hbar\omega_k}, \tag{SI.2a}$$

$$Z^\ddagger_{\mathrm{vib}} = \prod_{k=1}^{N_\mathrm{vib}-1} \frac{1}{2\sinh\frac{1}{2}\beta\hbar\tilde{\omega}_k}, \tag{SI.2b}$$

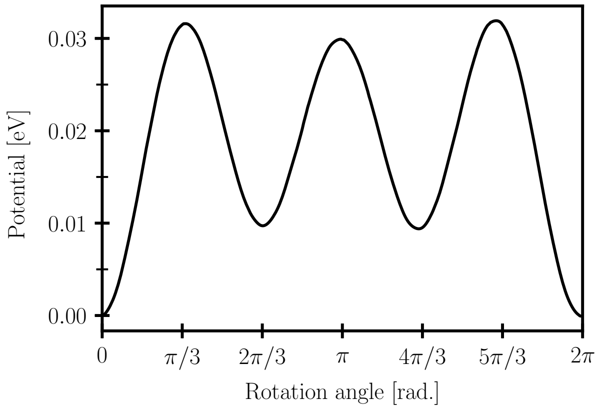

Figure SI.2: Potential-energy profile along the rigid rotation around the C–C bond, where the origin corresponds to the geometry of the T_1 minimum.

for the vibrational partition functions. Alternatively (but equivalently), one can define the partition functions

$$\widetilde{Z}_{\mathrm{T,vib}} = \prod_{k=1}^{N_{\mathrm{vib}}} \left(1 - \mathrm{e}^{-\beta\hbar\omega_k}\right)^{-1}, \qquad \text{(SI.3a)}$$

$$\widetilde{Z}^{\ddagger}_{\mathrm{vib}} = \prod_{k=1}^{N_{\mathrm{vib}}-1} \left(1 - \mathrm{e}^{-\beta\hbar\tilde{\omega}_k}\right)^{-1}, \qquad \text{(SI.3b)}$$

which measure the energy from the ZPE and are related to the formulation in Eqs. (SI.2) by $Z^{\ddagger}_{\mathrm{vib}}/Z_{\mathrm{T,vib}} = \mathrm{e}^{\beta\Delta\mathrm{ZPE}}\,\widetilde{Z}^{\ddagger}_{\mathrm{vib}}/\widetilde{Z}_{\mathrm{T,vib}}$, where $\Delta\mathrm{ZPE} = \frac{1}{2}\hbar[\sum_{k=1}^{N_{\mathrm{vib}}}\omega_k - \sum_{k=1}^{N_{\mathrm{vib}}-1}\tilde{\omega}_k]$ is the difference in zero-point energy between reactant and MECP. The NA-TST formula in terms of the alternative partition functions from Eqs. (SI.3) is then given by

$$k_{\text{NA-TST}}(\beta) = \sqrt{\frac{2\pi m}{\beta\hbar^2}}\,\frac{\Delta^2}{\hbar|\kappa_{\mathrm{T}} - \kappa_{\mathrm{S}}|}\,\frac{\widetilde{Z}^{\ddagger}}{\widetilde{Z}_{\mathrm{T}}}\,\mathrm{e}^{-\beta V^{\ddagger}_{\mathrm{ZPE}}}, \qquad \text{(SI.4)}$$

where $V^{\ddagger}_{\mathrm{ZPE}} = V^{\ddagger} - \Delta\mathrm{ZPE}$ is the ZPE-corrected barrier height.

In contrast to what was done in Ref. 24, we do not explicitly divide our rate constants by three to account for spin degeneracy. The fact that there are three triplet states and only one singlet state is already implicitly accounted for in the "effective" spin–orbit coupling, Δ.[29]

The NA-TST rate of Eq. (SI.1) completely neglects tunnelling, but includes zero-point energy effects and is therefore similar in spirit to Eyring transition-state theory for adiabatic reactions. The fully classical rate constant, which is also depicted in Fig. 2, is obtained by replacing the vibrational partition functions in Eq. (SI.1) by their classical ($\beta\hbar \to 0$) limits.

Building on NA-TST, the WC method approximates the tunnelling by effectively replacing the PESs by linear functions around the MECP along the singlet and triplet gradients. It then employs the quantum-mechanical solution for a system of two intersecting linear potentials leading to the WC transmission probability[30–32]

$$P_{\mathrm{WC}}(E) = 4\pi^2\sqrt{2m\beta_0}\,\frac{\Delta^2}{\hbar|\kappa_{\mathrm{S}} - \kappa_{\mathrm{T}}|} \times \mathrm{Ai}^2\left(\beta_0(V^{\ddagger}_{\mathrm{ZPE}} - E)\right), \qquad \text{(SI.5)}$$

where $\beta_0^3 = \frac{2m}{\hbar^2}(\kappa_{\mathrm{T}}^{-1} - \kappa_{\mathrm{S}}^{-1})^2$. The total WC rate constant is then given by:[29]

$$k_{\mathrm{WC}}(\beta) = \frac{1}{2\pi\hbar}\,\frac{\widetilde{Z}^{\ddagger}}{\widetilde{Z}_{\mathrm{T}}}\int_0^{\infty} P_{\mathrm{WC}}(E)\,\mathrm{e}^{-\beta E}\,\mathrm{d}E. \qquad \text{(SI.6)}$$

Because of the fundamental inconsistency between the linear approximation of the potentials around the MECP and the harmonic approximation around the reactant minimum there is no rigorous way to define the lower integration boundary in Eq. (SI.6) and hence several variations of the WC method have been used in the literature.[24,29,33] To enable comparison with previous work, here we use the WC method as originally applied to the cyclization reaction in Ref. 24. As we have shown elsewhere, none of the other choices can fix the fundamental problems of WC either.[12,34]

The one-dimensional WKB approximation to Fermi's golden rule, which assumes tunnelling takes place along the mass-weighted MEPs, is evaluated using

$$k_{\mathrm{WKB}}(\beta) = \frac{\sum_n k_{\mathrm{WKB}}(E_n)\,\mathrm{e}^{-\beta E_n}}{\sum_n \mathrm{e}^{-\beta E_n}}, \qquad \text{(SI.7)}$$

where $E_n = (n+\frac{1}{2})\hbar\omega_{\mathrm{rxc}}$ and the microcanonical WKB rate constants are given below. Here ω_{rxc} is the frequency of the reactive mode, which determines the curvature of the MEP near the reactant well in Fig. 1. For the cyclization this is found to be the lowest vibrational frequency (as is typically the case),[35] whereas in the isomerization reaction the lowest vibrational mode corresponds to a rotation of the CF_3 group around the C−C bond and does not participate significantly in the reaction. The appropriate ω_{rxc} thus corresponds to the second-lowest vibrational frequency. In the WKB⊥ method, which has also been used in Fig. 2, we also include the partition functions in the orthogonal modes giving:

$$k_{\mathrm{WKB}\perp}(\beta) = \frac{Z^{\ddagger}}{Z_{\mathrm{T}}} \sum_n k_{\mathrm{WKB}}(E_n)\, \mathrm{e}^{-\beta E_n}, \quad \text{(SI.8)}$$

where we used the vibrational partition functions from Eqs. (SI.2) to account for the ZPE.

At energies above the MECP, the microcanonical WKB golden-rule rate constant is[36]

$$\begin{aligned} k_{\mathrm{WKB}}(E) &= 4\omega_{\mathrm{rxc}}\gamma \sin^2\left(\frac{|W(E)|}{2\hbar} + \frac{\pi}{4}\right), \\ &\approx 2\omega_{\mathrm{rxc}}\gamma, \end{aligned} \quad \text{(SI.9)}$$

and below the MECP is given by[30,37–39]

$$k_{\mathrm{WKB}}(E) = \omega_{\mathrm{rxc}}\gamma\, \mathrm{e}^{-W(E)/\hbar}. \quad \text{(SI.10)}$$

For this one-dimensional problem, the total abbreviated action is $W(E) = W_{\mathrm{T}}(E) + W_{\mathrm{S}}(E)$ and $W_{\mathrm{T/S}}(E) = 2\int_a^b \sqrt{2m(V_{\mathrm{T/S}}(q) - E)}\,\mathrm{d}q$, where $\sqrt{m}q$ is the mass-weighted distance along the MEP. For the path on the triplet, the integral is from the turning point to the crossing point, while the integral for the path on the singlet surface is from the crossing point to the turning point. Finally, the Landau–Zener factor in the golden-rule limit is given by

$$\gamma = \frac{\Delta^2}{\hbar|\kappa_{\mathrm{T}} - \kappa_{\mathrm{S}}|v}, \quad \text{(SI.11)}$$

where the (absolute value of the) velocity is $v = \sqrt{2|V^{\ddagger} - E|/m}$.

3 Ring-polymer instanton results

In ring-polymer instanton theory, just as in other discretized path-integral based methods,[40] the results need to be converged with respect to the number of discretization points, or beads, of the path. A measure for how fine a chosen discretization is at a certain temperature is given by β/N, where N is the number of beads. This is due to the Trotter splitting used in the derivation of the methods, which introduces an error on the order of $(\beta/N)^2$.[41] The number of beads necessary for converging the results thus grows with decreasing temperature, i.e. if one wants to compute an instanton at half the temperature, one needs roughly twice as many beads to obtain the same quality of convergence. Note however that the required number of beads for convergence at a given temperature is system-dependent.

The convergence behaviour of the rate constant at various temperatures can be seen in Tables SI.5 and SI.6. We start our analysis with the simpler isomerization reaction (Table SI.6), for which it can be seen that the results at 30 K and higher temperatures are already well-converged with 256 beads, because the change in the rate constant upon doubling the number of beads is lower than 5%. However, as indicated by the results at 15 K, one requires at least 512 beads at lower temperatures to converge the result to the same quality. Furthermore, the low-temperature plateau has already been reached, since the results at the lowest temperatures are practically temperature-independent (within the numerical errors). It is therefore unnecessary to explicitly compute rate constants at even lower temperatures, which would require more beads just to obtain the same result.

In the cyclization reaction (Table SI.5), it becomes evident that the rate constants change more strongly with the number of beads. It is clear that the prefactor is responsible for this behaviour, as the actions are well behaved even when the overall result is far from its converged value. Nevertheless, it can be seen that the

Table SI.5: Convergence of the instanton actions and rate constants with respect to the number of beads for the cyclization reaction. Rate constants given in bold are considered converged to within 15% with respect to the number of beads.

T [K]	$S/\hbar$			k_{SCI} [$10^{-3}\,\mathrm{s}^{-1}$]		
	256	512	1024	256	512	1024
500	–	8.8466	–	–	$\mathbf{5.91 \cdot 10^9}$	–
300	–	13.7416	–	–	$\mathbf{5.63 \cdot 10^7}$	–
200	–	18.6392	–	–	$\mathbf{4.97 \cdot 10^5}$	–
100	26.5139	26.5118	–	206	**177**	–
50	29.3900	29.3802	29.3783	68.3	**11.6**	**10.3**
40	29.5340	29.5121	29.5138	535	**9.6**	**8.8**
30	–	29.5410	29.5530	–	**8.0**	**8.4**
25	–	29.5356	29.5497	–	**9.8**	**8.8**
20	–	29.5260	29.5397	–	7.4	**9.3**
15	–	29.5147	29.5249	–	4.4	**8.3**
10	–	29.4821	29.4930	–	86.5	7.0

rate constants between 25 K and 50 K computed with 512 beads are converged to within 15% of the results with 1024 beads. We can thus safely assume that calculations at higher temperatures will also be well-converged with the same number of beads (as they have an even lower β/N ratio). In order to find out when the low-temperature plateau is reached, one should compare rate constants with identical values of the β/N ratio (i.e. with the same quality of convergence) and see whether they are the same. This is analogous to the "diagonal convergence pattern" seen in instanton calculations of tunnelling splittings, as described in Ref. 42. In Table SI.5 we can therefore compare rate constants at 50 K and 25 K, 40 K and 20 K, 30 K and 15 K and 20 K and 10 K computed with 512 and 1024 beads respectively. While the values at 50 K and 25 K still differ significantly, the other pairs are almost identical, from which we can deduce that the low-temperature plateau has been reached at 40 K.[43] This is confirmed by the instanton energy in Table SI.7, which at 40 K drops to almost 0.[44] It is therefore the sensible choice to use the result with 1024 beads at 40 K also for lower temperatures, because it is expected to be the most accurate estimate of the low-temperature plateau as it has the lowest β/N ratio.

In Tables SI.7–SI.10 we present important quantities available from the instantons computed at various temperatures (obtained with the highest number of beads available). The Landau–Zener factor is evaluated from Eq. (SI.11), where the gradients and the barrier height are computed at the instanton hopping points and the instanton energy $E_{\mathrm{I}} = \partial S_{\mathrm{S}}/\partial\tau = -\partial S_{\mathrm{T}}/\partial\tau$ is taken to compute the imaginary velocity. In all cases, γ is seen to be much smaller than 1. Following the standard prescription,[35] this indicates that the two rates are very well approximated by their golden-rule limit. If this were not the case, it would, in principle, also be possible to compute higher orders in the perturbation series.[45]

Note also how at high temperatures the ratio $\tau/\beta\hbar$ converges to λ computed at the MECP, while at low temperatures the value of τ itself becomes constant indicating that the low-temperature plateau has been reached. This characteristic can be used to get an idea about whether the WC method will give reliable results for a system at a certain temperature. Thus, if instanton theory predicts a $\tau/\beta\hbar$ which is constant with temperature and close to λ from the MECP optimization, the WC approximation is likely to be valid. If, however, τ itself is constant, the WC results will be qualitatively

Table SI.6: Convergence of the instanton actions and rate constants with respect to the number of beads for the isomerization reaction. Rate constants given in bold are considered converged to within 5% with respect to the number of beads.

T [K]	$S/\hbar$			k_{SCI} [$10^{-3}\,\mathrm{s}^{-1}$]		
	256	512	1024	256	512	1024
500	14.1115	14.1113	–	**$8.622 \cdot 10^{7}$**	**$8.526 \cdot 10^{7}$**	–
300	21.0512	21.0506	–	**$1.109 \cdot 10^{5}$**	**$1.130 \cdot 10^{5}$**	–
200	26.3628	26.3613	–	**699.4**	**701.9**	–
100	30.4190	30.4139	–	**15.42**	**15.54**	–
50	30.7305	30.7208	–	**11.81**	**11.96**	–
30	30.7718	30.7361	30.7291	**11.39**	**11.29**	**11.59**
23	–	30.7470	30.7336	–	**11.28**	**11.49**
19	–	30.7672	30.7403	–	**11.54**	**11.60**
15	30.8760	30.7996	30.7524	12.81	**12.15**	**11.55**
11	–	30.8343	30.7869	–	**11.38**	**11.79**

Table SI.7: Data obtained from the instanton optimizations for the cyclization reaction. The action and instanton energy E_{I} are given with respect to the potential of the reactant minimum $V_{\mathrm{T}}^{\mathrm{min}}$. In addition to the rate constants based purely on the double-hybrid DFT calculations, we also give results corrected by the MRMP2//DFT barrier as described in the main text. Here, we defined $A = \sqrt{2\pi}\,\frac{\beta}{\hbar}\frac{Z_{\mathrm{vib}}^{\ddagger}}{Z_{\mathrm{T,vib}}}$, which includes the prefactor from Eq. (1) of the main text.

T [K]	A [a.u.]	$Z_{\mathrm{rot}}^{\ddagger}/Z_{\mathrm{T,rot}}$	$S/\hbar - \beta V_{\mathrm{T}}^{\mathrm{min}}$	E_{I} [cm^{-1}]	$\tau/\beta\hbar$	τ [a.u.]	γ	k_{SCI} [s^{-1}]	$k_{\mathrm{SCI}}^{\mathrm{MRMP2//DFT}}$ [s^{-1}]
500	44.4851	0.9535	8.8466	2781.6	0.4221	266.6	$3.5056 \cdot 10^{-4}$	$5.9065 \cdot 10^{6}$	$1.9101 \cdot 10^{6}$
400	51.2908	0.9538	10.7865	2609.0	0.3995	420.5	$2.9952 \cdot 10^{-4}$	$9.7907 \cdot 10^{5}$	$2.5347 \cdot 10^{5}$
300	56.6109	0.9545	13.7416	2322.6	0.4143	327.1	$2.4861 \cdot 10^{-4}$	$5.6311 \cdot 10^{4}$	$1.0634 \cdot 10^{4}$
250	60.8204	0.9560	15.8606	2097.1	0.3865	488.1	$2.2278 \cdot 10^{-4}$	$7.2805 \cdot 10^{3}$	$1.1209 \cdot 10^{3}$
200	66.7003	0.9574	18.6392	1767.5	0.3655	577.0	$1.9628 \cdot 10^{-4}$	$4.9675 \cdot 10^{2}$	$6.0804 \cdot 10^{1}$
150	68.7144	0.9597	22.2630	1267.7	0.3290	692.5	$1.6961 \cdot 10^{-4}$	$1.3688 \cdot 10^{1}$	$1.3643 \cdot 10^{0}$
125	67.4242	0.9616	24.3679	937.5	0.3005	759.2	$1.5690 \cdot 10^{-4}$	$1.6399 \cdot 10^{0}$	$1.5563 \cdot 10^{-1}$
100	61.8299	0.9644	26.5118	571.7	0.2618	826.6	$1.4566 \cdot 10^{-4}$	$1.7676 \cdot 10^{-1}$	$1.7260 \cdot 10^{-2}$
50	62.6471	0.9788	29.3783	34.2	0.1453	917.4	$1.3271 \cdot 10^{-4}$	$1.0342 \cdot 10^{-2}$	$1.3600 \cdot 10^{-3}$
40	60.7956	0.9829	29.5138	6.9	0.1168	922.0	$1.3215 \cdot 10^{-4}$	$8.8012 \cdot 10^{-3}$	$1.2066 \cdot 10^{-3}$
30	59.9865	0.9872	29.5530	0.0	0.0877	923.3	$1.3200 \cdot 10^{-4}$	$8.3859 \cdot 10^{-3}$	$1.1686 \cdot 10^{-3}$
25	62.8784	0.9886	29.5497	0.0	0.0731	923.4	$1.3200 \cdot 10^{-4}$	$8.8327 \cdot 10^{-3}$	$1.2342 \cdot 10^{-3}$

wrong.

In Tables SI.11 and SI.12, we show the contributions to the squared mass-weighted tunnelling path length (SMWTPL) from different atoms and atom groups at two different temperatures. While the tunnelling pathways in general become shorter at high temperatures, it can be seen that the significant relative contributions of the heavier nitrogen and oxygen atoms at cryogenic temperatures decrease at higher temperatures in favour of a larger relative contribution from the carbon atoms.

In Tables SI.13 and SI.14 we show KIEs calculated from the ratio of the (uncorrected) double-hybrid DFT rate constants, since the MRMP2 correction makes almost no difference here. The Tables contain KIEs computed based on independent instanton calculations with different masses and KIEs calculated from the change to the action upon isotopic substitution only, without reoptimization of the pathway. From the tables it can be seen that both approaches are in reasonably good agreement, which justifies the ap-

Table SI.8: As Table SI.7 but for the isomerization reaction. Here, the MRMP2 correction is with respect to the barrier obtained from the MRMP2 optimization of the MECP.

T [K]	A [a.u.]	$Z^{\ddagger}_{\mathrm{rot}}/Z_{\mathrm{T,rot}}$	$S/\hbar - \beta V_{\mathrm{T}}^{\mathrm{min}}$	E_{I} [cm^{-1}]	$\tau/\beta\hbar$	τ [a.u.]	γ	k_{SCI} [s^{-1}]	$k_{\mathrm{SCI}}^{\mathrm{MRMP2}}$ [s^{-1}]
500	251.7654	0.9833	14.1113	4248.5	0.4586	289.6	$8.8636\cdot10^{-5}$	$8.5264\cdot10^{4}$	$1.7318\cdot10^{4}$
450	271.3257	0.9829	15.4382	4048.0	0.4570	320.7	$8.0914\cdot10^{-5}$	$2.4371\cdot10^{4}$	$4.3822\cdot10^{3}$
400	291.9869	0.9825	17.0041	3784.4	0.4545	358.8	$7.3283\cdot10^{-5}$	$5.4766\cdot10^{3}$	$8.6013\cdot10^{2}$
350	313.4728	0.9821	18.8583	3435.2	0.4507	406.6	$6.5849\cdot10^{-5}$	$9.2024\cdot10^{2}$	$1.2528\cdot10^{2}$
300	344.8766	0.9818	21.0506	2967.1	0.4442	467.5	$5.8691\cdot10^{-5}$	$1.1093\cdot10^{2}$	$1.3362\cdot10^{1}$
250	376.9534	0.9817	23.5929	2341.8	0.4322	545.9	$5.1978\cdot10^{-5}$	$9.7016\cdot10^{0}$	$1.0311\cdot10^{0}$
200	433.4205	0.9826	26.3613	1543.4	0.4078	643.9	$4.6054\cdot10^{-5}$	$6.9941\cdot10^{-1}$	$7.2720\cdot10^{-2}$
150	487.8484	0.9850	28.9135	679.6	0.3561	749.7	$4.1537\cdot10^{-5}$	$6.1708\cdot10^{-2}$	$7.2278\cdot10^{-3}$
125	522.6874	0.9874	29.8418	330.0	0.3138	792.8	$4.0097\cdot10^{-5}$	$2.6192\cdot10^{-2}$	$3.4247\cdot10^{-3}$
100	548.1312	0.9897	30.4139	109.1	0.2598	820.4	$3.9287\cdot10^{-5}$	$1.5536\cdot10^{-2}$	$2.2427\cdot10^{-3}$
50	569.9121	0.9950	30.7208	1.2	0.1321	834.3	$3.8941\cdot10^{-5}$	$1.1949\cdot10^{-2}$	$1.8574\cdot10^{-3}$
40	556.9260	0.9960	30.7263	0.3	0.1057	834.5	$3.8940\cdot10^{-5}$	$1.1624\cdot10^{-2}$	$1.8082\cdot10^{-3}$
30	556.2522	0.9971	30.7291	0.2	0.0793	834.5	$3.8938\cdot10^{-5}$	$1.1159\cdot10^{-2}$	$1.8031\cdot10^{-3}$
23	553.5830	0.9976	30.7336	0.2	0.0608	834.5	$3.8939\cdot10^{-5}$	$1.1489\cdot10^{-2}$	$1.7863\cdot10^{-3}$
19	562.7572	0.9979	30.7403	0.2	0.0502	834.5	$3.8941\cdot10^{-5}$	$1.1604\cdot10^{-2}$	$1.8025\cdot10^{-3}$
15	566.6550	0.9982	30.7524	0.2	0.0396	834.5	$3.8941\cdot10^{-5}$	$1.1548\cdot10^{-2}$	$1.7910\cdot10^{-3}$
11	598.4587	0.9988	30.7869	0.3	0.0291	834.8	$3.8941\cdot10^{-5}$	$1.1790\cdot10^{-2}$	$1.8210\cdot10^{-3}$
10	606.9628	0.9989	30.7865	0.2	0.0264	834.8	$3.8942\cdot10^{-5}$	$1.1964\cdot10^{-2}$	$1.8475\cdot10^{-3}$

Table SI.9: As Table SI.7 but with ^{15}N.

T [K]	A [a.u.]	$Z^{\ddagger}_{\mathrm{rot}}/Z_{\mathrm{T,rot}}$	$S/\hbar - \beta V_{\mathrm{T}}^{\mathrm{min}}$	E_{I} [cm^{-1}]	$\tau/\beta\hbar$	τ [a.u.]	γ	k_{SCI} [s^{-1}]	$k_{\mathrm{SCI}}^{\mathrm{MRMP2//DFT}}$ [s^{-1}]
30	59.1615	0.9868	29.8971	0.0	0.0888	934.8	$1.3288\cdot10^{-4}$	$5.8607\cdot10^{-3}$	$7.9853\cdot10^{-4}$
25	64.3420	0.9887	29.9068	0.0	0.0740	934.8	$1.3285\cdot10^{-4}$	$6.3245\cdot10^{-3}$	$8.6285\cdot10^{-4}$

proximate calculation. This method is similar to the one employed in Ref. 46 and can be seen as a perturbative approach in the following sense. We define the action along a stationary path $\mathsf{x}(\boldsymbol{\alpha}_0)$ with imaginary-time split $\tau(\boldsymbol{\alpha}_0)$ as $S(\mathsf{x}(\boldsymbol{\alpha}_0), \tau(\boldsymbol{\alpha}_0); \boldsymbol{\alpha}_0)$, where $\boldsymbol{\alpha}_0$ is a vector of reference values of a parameter (e.g. the atomic masses or the potential energy). If $\boldsymbol{\alpha}$ is the vector of the new parameter values (e.g. the masses after isotopic substitution or the potentials after correction by a higher-level electronic-structure method) then the new action is:

$$\begin{aligned} S(\mathsf{x}(\boldsymbol{\alpha}), \tau(\boldsymbol{\alpha}); \boldsymbol{\alpha}) &= S(\mathsf{x}(\boldsymbol{\alpha}_0), \tau(\boldsymbol{\alpha}_0); \boldsymbol{\alpha}_0) + (\boldsymbol{\alpha} - \boldsymbol{\alpha}_0) \cdot \left[\frac{\partial S}{\partial \mathsf{x}} \frac{\partial \mathsf{x}}{\partial \boldsymbol{\alpha}} + \frac{\partial S}{\partial \tau} \frac{\partial \tau}{\partial \boldsymbol{\alpha}} + \frac{\partial S}{\partial \boldsymbol{\alpha}} \right]_{\boldsymbol{\alpha}=\boldsymbol{\alpha}_0} + \mathcal{O}(\boldsymbol{\alpha} - \boldsymbol{\alpha}_0)^2, \\ &= S(\mathsf{x}(\boldsymbol{\alpha}_0), \tau(\boldsymbol{\alpha}_0); \boldsymbol{\alpha}_0) + (\boldsymbol{\alpha} - \boldsymbol{\alpha}_0) \cdot \left[\frac{\partial S}{\partial \boldsymbol{\alpha}} \right]_{\boldsymbol{\alpha}=\boldsymbol{\alpha}_0} + \mathcal{O}(\boldsymbol{\alpha} - \boldsymbol{\alpha}_0)^2, \\ &= S(\mathsf{x}(\boldsymbol{\alpha}_0), \tau(\boldsymbol{\alpha}_0); \boldsymbol{\alpha}) + \mathcal{O}(\boldsymbol{\alpha} - \boldsymbol{\alpha}_0)^2, \end{aligned} \tag{SI.12}$$

where we used $\frac{\partial S}{\partial \mathsf{x}}|_{\boldsymbol{\alpha}=\boldsymbol{\alpha}_0} = \mathsf{0}$ and $\frac{\partial S}{\partial \tau}|_{\boldsymbol{\alpha}=\boldsymbol{\alpha}_0} = 0$. This demonstrates that our simple approximation (which involves recomputing the action with the new parameters along the reference pathway) is correct to first order in $(\boldsymbol{\alpha} - \boldsymbol{\alpha}_0)$ at least. This provides formal justification for the approach used for estimating the KIE as well as correcting the barrier height with the MRMP2 energy.

Table SI.10: As Table SI.8 but with ^{15}N.

T [K]	A [a.u.]	$Z^{\ddagger}_{\mathrm{rot}}/Z_{\mathrm{T,rot}}$	$S/\hbar - \beta V_{\mathrm{T}}^{\mathrm{min}}$	E_{I} [cm^{-1}]	$\tau/\beta\hbar$	τ [a.u.]	γ	k_{SCI} [s^{-1}]	$k_{\mathrm{SCI}}^{\mathrm{MRMP2}}$ [s^{-1}]
500	255.3337	0.9813	14.1285	4264.0	0.4589	289.8	$9.0227\cdot10^{-5}$	$8.4830\cdot10^{4}$	$1.7194\cdot10^{4}$
300	347.7550	0.9799	21.1107	2995.7	0.4449	468.3	$5.9659\cdot10^{-5}$	$1.0709\cdot10^{2}$	$1.2535\cdot10^{1}$
200	442.1199	0.9808	26.5015	1578.8	0.4094	646.4	$4.6743\cdot10^{-5}$	$6.2118\cdot10^{-1}$	$6.2938\cdot10^{-2}$
100	569.7159	0.9884	30.7221	118.6	0.2628	829.8	$3.9726\cdot10^{-5}$	$1.1851\cdot10^{-2}$	$1.6673\cdot10^{-3}$
50	595.4370	0.9944	31.0636	1.3	0.1339	845.5	$3.9348\cdot10^{-5}$	$8.8553\cdot10^{-3}$	$1.3501\cdot10^{-3}$
40	597.7469	0.9955	31.0740	0.1	0.1071	845.8	$3.9346\cdot10^{-5}$	$8.8085\cdot10^{-3}$	$1.3436\cdot10^{-3}$
30	584.4757	0.9967	31.0707	0.2	0.0803	845.7	$3.9347\cdot10^{-5}$	$8.6512\cdot10^{-3}$	$1.3208\cdot10^{-3}$
23	589.1382	0.9973	31.0788	0.2	0.0616	845.8	$3.9348\cdot10^{-5}$	$8.6550\cdot10^{-3}$	$1.3200\cdot10^{-3}$
19	599.0141	0.9976	31.0861	0.2	0.0509	845.8	$3.9346\cdot10^{-5}$	$8.7389\cdot10^{-3}$	$1.3315\cdot10^{-3}$
15	594.4640	0.9984	31.0998	0.2	0.0402	846.0	$3.9347\cdot10^{-5}$	$8.5605\cdot10^{-3}$	$1.3024\cdot10^{-3}$
11	602.2554	0.9987	31.1221	0.2	0.0291	846.0	$3.9347\cdot10^{-5}$	$8.4849\cdot10^{-3}$	$1.2870\cdot10^{-3}$
10	602.1899	0.9989	31.1343	0.2	0.0268	846.3	$3.9344\cdot10^{-5}$	$8.3828\cdot10^{-3}$	$1.2700\cdot10^{-3}$

Table SI.11: Relative contributions in % to the SMWTPL per atom or atom group for the cyclization reaction at different temperatures. The contributions of the hydrogen atoms have been combined. The carbon atoms have been labelled as either α or β; the latter set includes the three carbons in the six-membered ring connected to hydrogen atoms, and the former set contains the remainder.

T [K]	Atoms					
	N	O	C_α	C_β	F	H
300	32	31	32	4	0	1
25	35	48	11	3	1	2

Table SI.12: As Table SI.11 but for the isomerization reaction. The α carbon corresponds to the one in the NCO group, while the β carbon is part of the CF_3 group. The contributions of the fluorine atoms have been combined.

T [K]	Atoms				
	N	O	C_α	C_β	F
300	26	4	55	12	3
15	34	9	43	7	7

Table SI.13: KIEs for the cyclization reaction calculated from the full rate expression, after reoptimization of the instanton, and from the approximation based on the change to the action only, without explicit reoptimization of the instanton pathway.

T [K]	^{14}N/^{15}N		^{16}O/^{18}O
	full	approx.	approx.
500	–	1.0	1.0
300	–	1.0	1.1
200	–	1.1	1.1
100	–	1.3	1.7
50	–	1.4	2.3
40	–	1.4	2.4
30	1.4	1.4	2.4
25	1.4	1.4	2.4

Table SI.14: As Table SI.13 but for the isomerization reaction. For the calculation of the $^{12}C/^{13}C$ KIE, only the α carbon in the NCO group has been isotopically substituted.

T [K]	$^{14}N/^{15}N$		$^{12}C/^{13}C$
	full	approx.	approx.
500	1.00	1.02	1.04
300	1.04	1.06	1.17
200	1.13	1.15	1.41
100	1.31	1.36	1.74
50	1.35	1.41	1.77
40	1.32	1.42	1.77
30	1.29	1.41	1.77
23	1.33	1.41	1.77
19	1.33	1.41	1.77
15	1.35	1.42	1.77

4 Collected results

The rate constants based purely on double-hybrid DFT computed with the various rate theories introduced before are depicted in Fig. SI.3. This differs from Fig. 2 of the main text, in which the MRMP2 correction is used.

To enable closer comparison, rate constants and KIEs computed with the various methods along with the experimental values for the two reactions are presented in Tables SI.15 and SI.16.

In the cyclization reaction, the WC method predicts no kinetic isotope effect (i.e. a KIE of 1.0). This is due to a coincidental cancellation between the effects of a slight increase in the mass-weighted barrier width and a slight decrease in the ZPE-corrected barrier height. The more reliable instanton results, however, predict that there is no such cancellation. In the isomerization reaction, the ZPE-corrected barrier height increases on isotopic substitution, such that there is no possibility of cancellation.

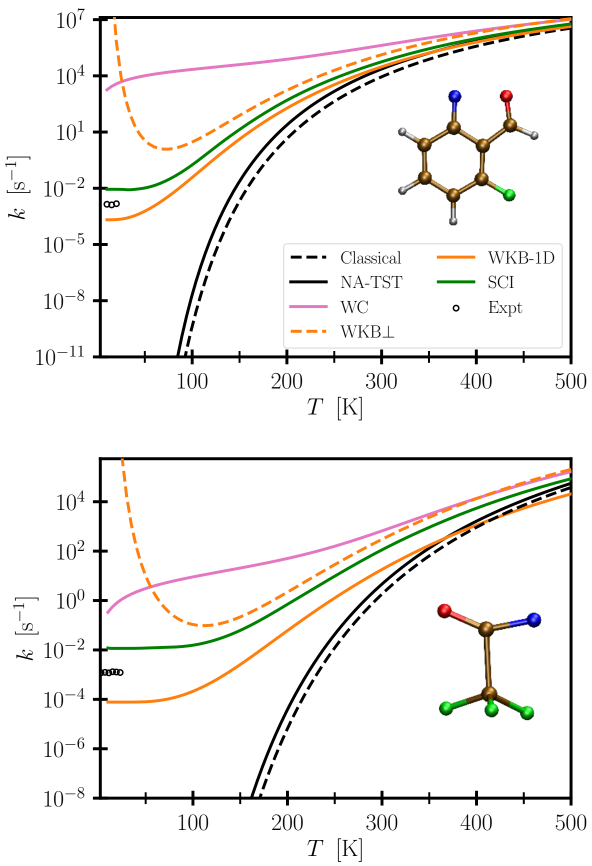

Figure SI.3: Uncorrected rate constants, using only data from double-hybrid DFT calculations, for the cyclization (top) and isomerization reaction (bottom) computed with various methods compared to experiment.[24,47] The insets show the molecular structure at the MECP of the respective reaction.

Table SI.15: Reaction rate constants (in $10^{-3}\,\mathrm{s}^{-1}$) and predicted $^{14}N/^{15}N$ KIEs for the cyclization reaction. We give NA-TST, WC and instanton results based on the double-hybrid DFT calculations and the values corrected by the MRMP2//DFT barrier as described in the text. At temperatures for which the rate constant is deemed to have plateaued, instantons are not explicitly calculated and instead the plateau values are indicated by parentheses.

T [K]	$k_{\text{NA-TST}}$	$k_{\text{NA-TST}}^{\text{MRMP2//DFT}}$	k_{WC}	$k_{\text{WC}}^{\text{MRMP2//DFT}}$	k_{SCI}	$k_{\text{SCI}}^{\text{MRMP2//DFT}}$	k_{exp}	KIE_{WC}	KIE_{SCI}
30	$6.5 \cdot 10^{-48}$	$5.4 \cdot 10^{-57}$	$5.9 \cdot 10^{6}$	$3.8 \cdot 10^{5}$	8.4	1.2	–	1.0	1.4
25	$5.7 \cdot 10^{-60}$	$5.9 \cdot 10^{-71}$	$4.8 \cdot 10^{6}$	$3.1 \cdot 10^{5}$	8.8	1.2	–	1.0	1.4
20	$2.9 \cdot 10^{-78}$	$7.0 \cdot 10^{-92}$	$3.8 \cdot 10^{6}$	$2.4 \cdot 10^{5}$	(8.8)	(1.2)	1.5	1.0	(1.4)
15	$1.4 \cdot 10^{-108}$	$9.5 \cdot 10^{-127}$	$2.8 \cdot 10^{6}$	$1.8 \cdot 10^{5}$	(8.8)	(1.2)	1.3	1.0	(1.4)
10	$3.3 \cdot 10^{-169}$	$1.9 \cdot 10^{-196}$	$1.8 \cdot 10^{6}$	$1.1 \cdot 10^{5}$	(8.8)	(1.2)	1.4	1.0	(1.4)

Table SI.16: Reaction rate constants (in $10^{-3}\,\mathrm{s}^{-1}$) and $^{14}N/^{15}N$ KIE for the isomerization reaction. We give NA-TST, WC and instanton results based on the double-hybrid DFT calculations and the values corrected by the MRMP2 barrier as described in the text. A range for the experimental results is given, where multiple measurements have been carried out. At temperatures for which the rate constant is deemed to have plateaued, instantons are not explicitly calculated and instead the plateau values are indicated by parentheses.

T [K]	$k_{\text{NA-TST}}$	$k_{\text{NA-TST}}^{\text{MRMP2}}$	k_{WC}	$k_{\text{WC}}^{\text{MRMP2}}$	k_{SCI}	$k_{\text{SCI}}^{\text{MRMP2}}$	k_{exp}	KIE_{WC}	KIE_{SCI}	KIE_{exp}
23	$2.82{\cdot}10^{-118}$	$9.98{\cdot}10^{-136}$	1020	18.8	11.5	1.79	1.22 – 1.78	1.21	1.33	–
19	$7.92{\cdot}10^{-146}$	$5.94{\cdot}10^{-167}$	779	14.4	11.6	1.80	1.21 – 1.30	1.21	1.33	–
15	$4.71{\cdot}10^{-188}$	$8.21{\cdot}10^{-215}$	564	10.4	11.6	1.79	1.31	1.21	1.35	–
11	$5.97{\cdot}10^{-261}$	$1.94{\cdot}10^{-297}$	376	6.95	(11.6)	(1.79)	1.18	1.21	(1.35)	–
7	$4.53{\cdot}10^{-417}$	$2.07{\cdot}10^{-474}$	219	4.04	(11.6)	(1.79)	1.24	1.21	(1.35)	1.32/1.18
2.8	$9.62{\cdot}10^{-1061}$	$4.31{\cdot}10^{-1204}$	83.7	1.54	(11.6)	(1.79)	1.19	1.22	(1.35)	1.24 – 1.44

References

(1) Grimme, S. Semiempirical hybrid density functional with perturbative second-order correlation. *J. Chem. Phys.* **2006**, *124*, 034108.

(2) Feyereisen, M.; Fitzgerald, G.; Komornicki, A. Use of approximate integrals in ab initio theory. An application in MP2 energy calculations. *Chem. Phys. Lett.* **1993**, *208*, 359–363.

(3) Hellweg, A.; Hättig, C.; Höfener, S.; Klopper, W. Optimized accurate auxiliary basis sets for RI-MP2 and RI-CC2 calculations for the atoms Rb to Rn. *Theor. Chem. Acc.* **2007**, *117*, 587–597.

(4) Neese, F. The ORCA program system. *WIREs Comput. Mol. Sci.* **2011**, *2*, 73–78.

(5) Neese, F. Software update: the ORCA program system, version 4.0. *WIREs Comput. Mol. Sci.* **2017**, *8*, e1327.

(6) Grimme, S.; Ehrlich, S.; Goerigk, L. Effect of the damping function in dispersion corrected density functional theory. *J. Comput. Chem.* **2011**, *32*, 1456–1465.

(7) Johnson, E. R.; Becke, A. D. A post-Hartree-Fock model of intermolecular interactions: Inclusion of higher-order corrections. *J. Chem. Phys.* **2006**, *124*, 174104.

(8) Weigend, F.; Ahlrichs, R. Balanced basis sets of split valence, triple zeta valence and quadruple zeta valence quality for H to Rn: Design and assessment of accuracy. *Phys. Chem. Chem. Phys.* **2005**, *7*, 3297.

(9) Weigend, F. Accurate Coulomb-fitting basis sets for H to Rn. *Phys. Chem. Chem. Phys.* **2006**, *8*, 1057.

(10) Weigend, F. Hartree–Fock exchange fitting basis sets for H to Rn. *J. Comput. Chem.* **2007**, *29*, 167–175.

(11) Koga, N.; Morokuma, K. Determination of the lowest energy point on the crossing seam between two potential surfaces using the energy gradient. *Chem. Phys. Lett.* **1985**, *119*, 371–374.

(12) Ansari, I. M.; Heller, E. R.; Trenins, G.; Richardson, J. O. Instanton theory for Fermi's golden rule and beyond. *Phil. Trans. R. Soc. A.* **2022**, *380*, 20200378.

(13) Wales, D. J. Locating stationary points for clusters in cartesian coordinates. *J. Chem. Soc., Faraday Trans.* **1993**, *89*, 1305–1313.

(14) Doye, J. P. K.; Wales, D. J. Saddle points and dynamics of Lennard-Jones clusters, solids, and supercooled liquids. *J. Chem. Phys.* **2002**, *116*, 3777–3788.

(15) Wales, D. J. *Energy Landscapes*; Cambridge University Press: Cambridge, 2003.

(16) Fletcher, R. *Practical Methods of Optimization*, 2nd ed.; Wiley: Chichester, 1987.

(17) Hirao, K. Multireference Møller—Plesset perturbation theory for high-spin open-shell systems. *Chem. Phys. Lett.* **1992**, *196*, 397–403.

(18) Hirao, K. Multireference Møller—Plesset method. *Chem. Phys. Lett.* **1992**, *190*, 374–380.

(19) Schmidt, M. W.; Baldridge, K. K.; Boatz, J. A.; Elbert, S. T.; Gordon, M. S.; Jensen, J. H.; Koseki, S.; Matsunaga, N.; Nguyen, K. A.; Su, S.; Windus, T. L.; Dupuis, M.; Montgomery, J. A. General atomic and molecular electronic structure system. *J. Comp. Chem.* **1993**, *14*, 1347–1363.

(20) Gordon, M. S.; Schmidt, M. W. In *Theory and Applications of Computational Chemistry: the first forty years*; Dykstra, C., Frenking, G., Kim, K., Scuseri, G., Eds.; Elsevier: Amsterdam, 2005; pp 1167–1189.

(21) Barca, G. M. J. et al. Recent developments in the general atomic and molecular electronic structure system. *J. Chem. Phys.* **2020**, *152*, 154102.

(22) Staroverov, V. N.; Scuseria, G. E.; Tao, J.; Perdew, J. P. Comparative assessment of a new nonempirical density functional: Molecules and hydrogen-bonded complexes. *J. Chem. Phys.* **2003**, *119*, 12129–12137.

(23) de Souza, B.; Farias, G.; Neese, F.; Izsák, R. Predicting Phosphorescence Rates of Light Organic Molecules Using Time-Dependent Density Functional Theory and the Path Integral Approach to Dynamics. *J. Chem. Theory Comput.* **2019**, *15*, 1896–1904.

(24) Nunes, C. M.; Viegas, L. P.; Wood, S. A.; Roque, J. P. L.; McMahon, R. J.; Fausto, R. Heavy-Atom Tunneling Through Crossing Potential Energy Surfaces: Cyclization of a Triplet 2-Formylarylnitrene to a Singlet 2,1-Benzisoxazole. *Angew. Chem. Int. Ed.* **2020**, *59*, 17622–17627.

(25) Harvey, J. N.; Aschi, M. Spin-forbidden dehydrogenation of methoxy cation: a statistical view. *Phys. Chem. Chem. Phys.* **1999**, *1*, 5555–5563.

(26) Lorquet, J. C.; Leyh-Nihant, B. Nonadiabatic unimolecular reactions. 1. A statistical formulation for the rate constants. *J. Phys. Chem.* **1988**, *92*, 4778–4783.

(27) Cui, Q.; Morokuma, K.; Bowman, J. M. The spin-forbidden reaction $CH(^2\Pi) + N_2 \rightarrow HCN + N(^4S)$ revisited. II. Nonadiabatic transition state theory and application. *J. Chem. Phys* **1999**, *110*, 9469.

(28) Harvey, J. N. Spin-forbidden reactions: computational insight into mechanisms and kinetics. *WIREs Comput. Mol. Sci.* **2014**, *4*, 1–14.

(29) Lykhin, A. O.; Kaliakin, D. S.; dePolo, G. E.; Kuzubov, A. A.; Varganov, S. A. Nonadiabatic transition state theory: Application to intersystem crossings in the active sites of metal-sulfur proteins. *Int. J. Quantum Chem.* **2016**, *116*, 750–761.

(30) Landau, L. D.; Lifshitz, E. M. *Quantum Mechanics: Non-Relativistic Theory*, 2nd ed.; Pergamon Press: Oxford, 1965.

(31) Delos, J. B. On the reactions of N_2 with O. *J. Chem. Phys.* **1973**, *59*, 2365–2369.

(32) Nikitin, E. E.; Umanskii, S. Y. *Theory of Slow Atomic Collisions*; Springer-Verlag, 1984.

(33) Lykhin, A. O.; Varganov, S. A. Intersystem crossing in tunneling regime: $T_1 \rightarrow S_0$ relaxation in thiophosgene. *Phys. Chem. Chem. Phys.* **2020**, *22*, 5500–5508.

(34) Heller, E. R.; Richardson, J. O. Spin Crossover of Thiophosgene via Multidimensional Heavy-Atom Quantum Tunneling. *J. Am. Chem. Soc.* **2021**, *143*, 20952–20961.

(35) Benderskii, V. A.; Goldanskii, V. I.; Makarov, D. E. Quantum dynamics in low-temperature chemistry. *Phys. Rep.* **1993**, *233*, 195–339.

(36) Child, M. S. *Semiclassical Mechanics with Molecular Applications*, 2nd ed.; Oxford, 2014.

(37) Child, M. Semi-classical analysis of weakly inelastic molecular collisions. *Mol. Phys.* **1964**, *8*, 517–531.

(38) Miller, W. H. Uniform semiclassical approximations for elastic scattering and eigenvalue problems. *J. Chem. Phys.* **1968**, *48*, 464–467.

(39) Ulstrup, J. *Charge Transfer Processes in Condensed Media*; Springer-Verlag: Berlin, 1979.

(40) Feynman, R. P.; Hibbs, A. R. *Quantum Mechanics and Path Integrals*; McGraw-Hill: New York, 1965.

(41) Tuckerman, M. E. *Statistical Mechanics: Theory and Molecular Simulation*; Oxford University Press: Oxford, 2010.

(42) Richardson, J. O.; Althorpe, S. C. Ring-polymer instanton method for calculating tunneling splittings. *J. Chem. Phys.* **2011**, *134*, 054109.

(43) From this analysis, we can additionally infer that the results at 20 K and 15 K are sufficiently converged with $N = 1024$ but that the result at 10 K is not.

(44) The fact that the instanton energy in the low-temperature limit does not always go down to exactly 0 is solely due to small numerical errors.

(45) Trenins, G.; Richardson, J. O. Nonadiabatic instanton rate theory beyond the golden-rule limit. *J. Chem. Phys.* **2022**, *156*, 174115.

(46) Meisner, J.; Rommel, J. B.; Kästner, J. Kinetic isotope effects calculated with the instanton method. *J. Comput. Chem.* **2011**, *32*, 3456–3463.

(47) Wu, Z.; Feng, R.; Li, H.; Xu, J.; Deng, G.; Abe, M.; Bégué, D.; Liu, K.; Zeng, X. Fast Heavy-Atom Tunneling in Trifluoroacetyl Nitrene. *Angew. Chem. Int. Ed.* **2017**, *56*, 15672–15676.
