# Supplementary figures and images for: Heavy‐Atom Quantum Tunnelling in Spin Crossovers of Nitrenes
Source: Angew Chem Int Ed Engl. 2022 Jul 5;61(33):e202206314. doi: 10.1002/anie.202206314 (PMC9540336; doi:10.1002/anie.202206314)

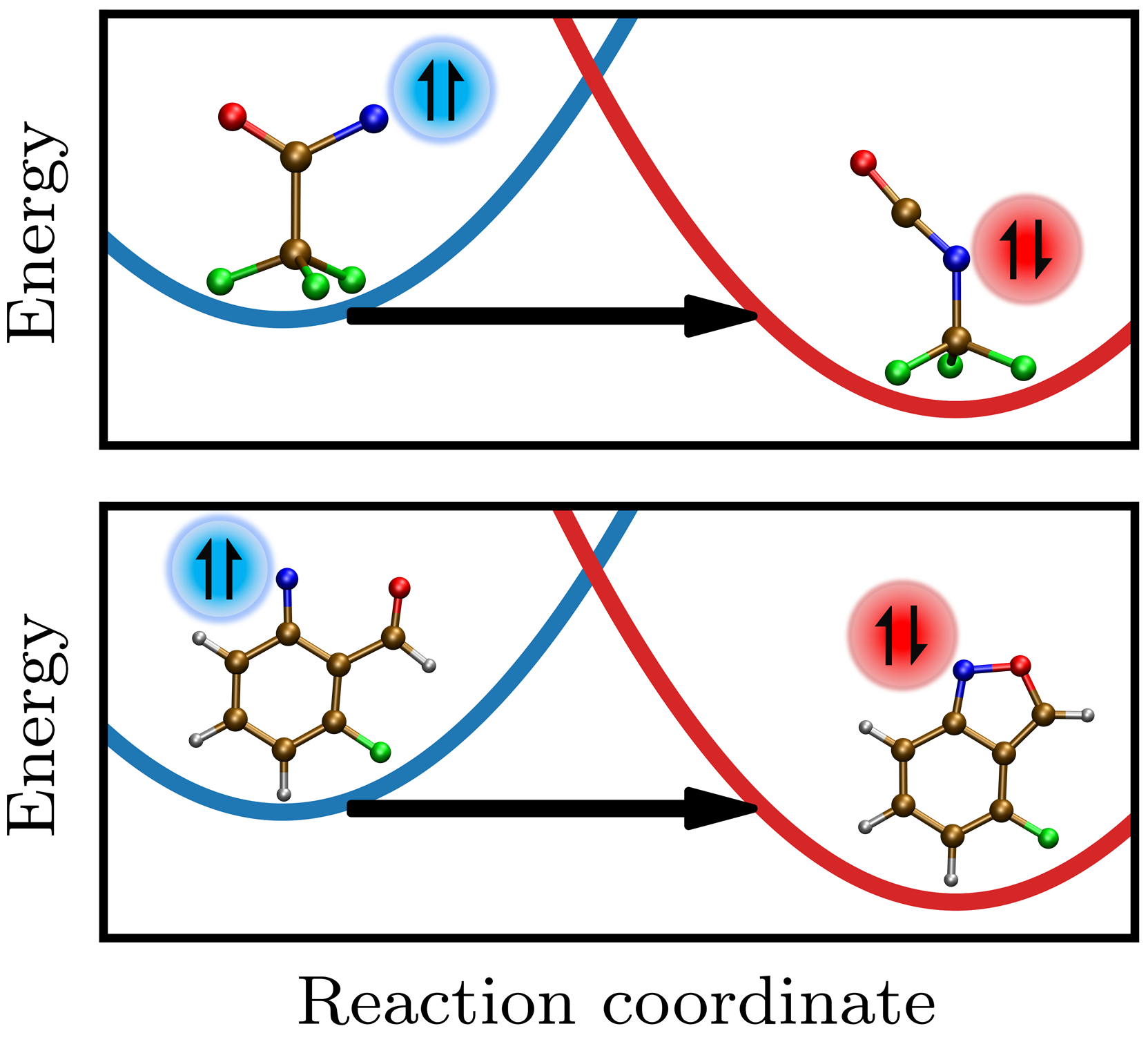

Supplement: Supplementary file 8 — Supporting Information [file ANIE-61-0-s006.png]

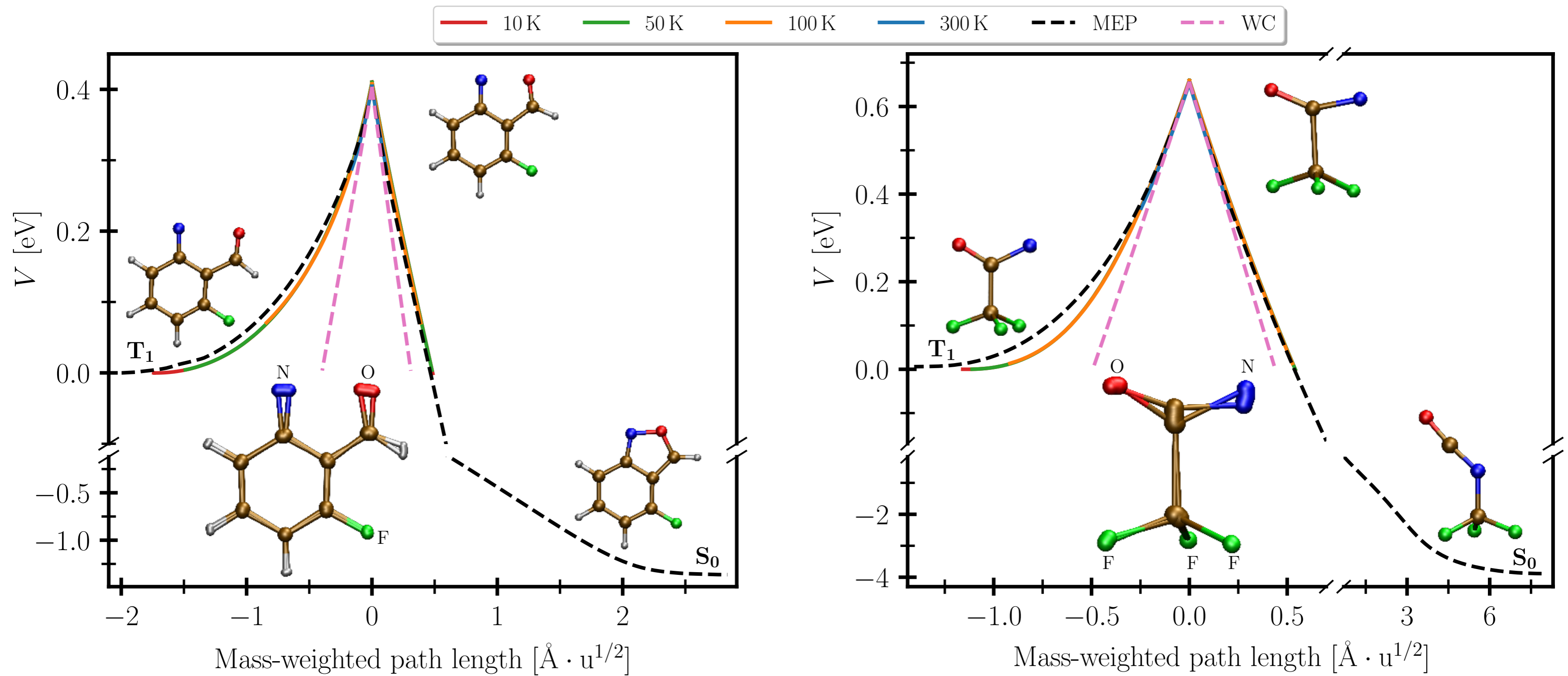

Supplement: Supplementary file 9 — Supporting Information [file ANIE-61-0-s008.pdf]

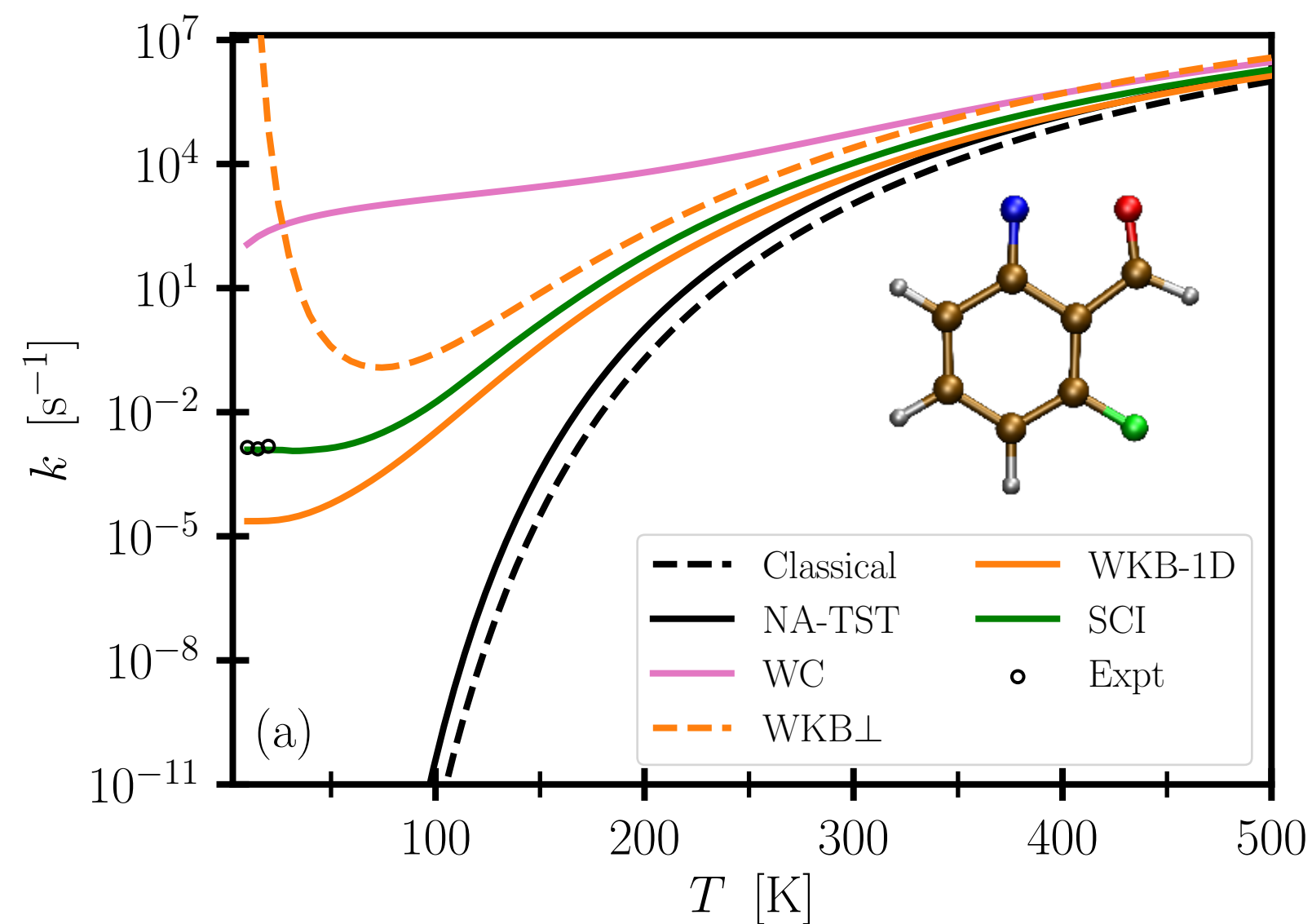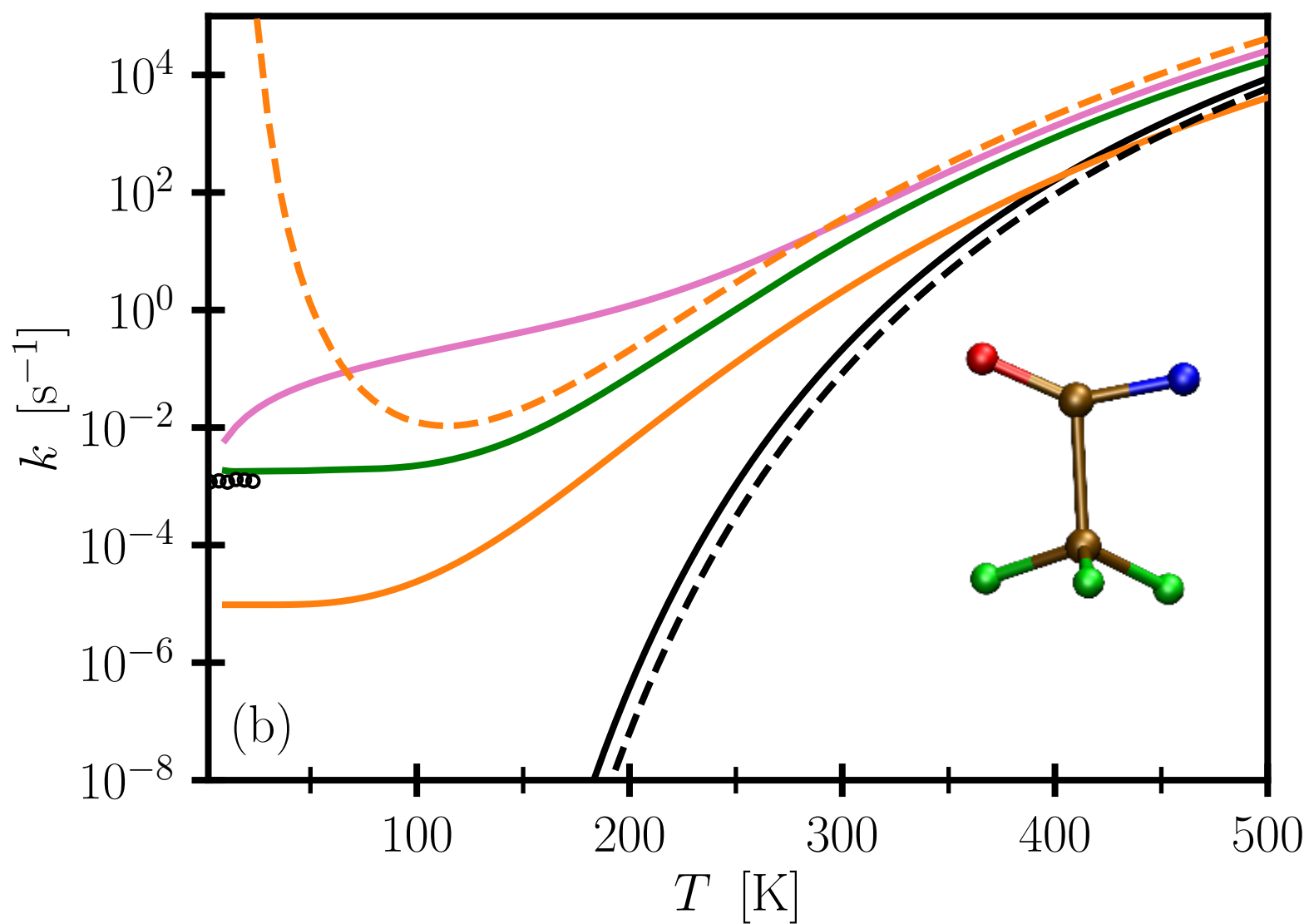

Supplement: Supplementary file 10 — Supporting Information [file ANIE-61-0-s005.pdf]
